# Development of Neuronal Guidance Fibers for Stimulating Electrodes: Basic Construction and Delivery of a Growth Factor

**DOI:** 10.3389/fbioe.2022.776890

**Published:** 2022-01-24

**Authors:** Inga Wille, Jennifer Harre, Sarah Oehmichen, Maren Lindemann, Henning Menzel, Nina Ehlert, Thomas Lenarz, Athanasia Warnecke, Peter Behrens

**Affiliations:** ^1^ Institut für Anorganische Chemie, Leibniz Universität Hannover, Hannover, Germany; ^2^ Cluster of Excellence Hearing4all, Hannover, Germany; ^3^ Department of Otorhinolaryngology, Head and Neck Surgery, Hannover Medical School, Hannover, Germany; ^4^ Institut für Technische Chemie, Technische Universität Braunschweig, Braunschweig, Germany; ^5^ Cluster of Excellence PhoenixD, Hannover, Germany

**Keywords:** cochlea implant, implant-associated drug delivery, BDNF, heparan sulfate, polycaprolactone, polyglycolide

## Abstract

State-of-the-art treatment for sensorineural hearing loss is based on electrical stimulation of residual spiral ganglion neurons (SGNs) with cochlear implants (CIs). Due to the anatomical gap between the electrode contacts of the CI and the residual afferent fibers of the SGNs, spatial spreading of the stimulation signal hampers focused neuronal stimulation. Also, the efficiency of a CI is limited because SGNs degenerate over time due to loss of trophic support. A promising option to close the anatomical gap is to install fibers as artificial nerve guidance structures on the surface of the implant and install on these fibers drug delivery systems releasing neuroprotective agents. Here, we describe the first steps in this direction. In the present study, suture yarns made of biodegradable polymers (polyglycolide/poly-ε-caprolactone) serve as the basic fiber material. In addition to the unmodified fiber, also fibers modified with amine groups were employed. Cell culture investigations with NIH 3T3 fibroblasts attested good cytocompatibility to both types of fibers. The fibers were then coated with the extracellular matrix component heparan sulfate (HS) as a biomimetic of the extracellular matrix. HS is known to bind, stabilize, modulate, and sustainably release growth factors. Here, we loaded the HS-carrying fibers with the brain-derived neurotrophic factor (BDNF) which is known to act neuroprotectively. Release of this neurotrophic factor from the fibers was followed over a period of 110 days. Cell culture investigations with spiral ganglion cells, using the supernatants from the release studies, showed that the BDNF delivered from the fibers drastically increased the survival rate of SGNs *in vitro*. Thus, biodegradable polymer fibers with attached HS and loaded with BDNF are suitable for the protection and support of SGNs. Moreover, they present a promising base material for the further development towards a future neuronal guiding scaffold.

## Introduction

According to the World Health Organization, in 2020 around 466 million people in the world have disabling hearing loss and it is estimated that by 2050 this number will rise to over 900 million people (WHO, 2021). The overall relevance of this sensory disorder is underlined by the fact that nowadays around 278 million people suffer from severe hearing loss so that normal activities, such as having a conversation, are not possible anymore without a hearing aid (Hearing loss is a big worldwide problem, 2016). Sensorineural hearing loss is the most common form of hearing loss and is caused by damage of the sensory hair cells within the cochlea. The insertion of a cochlear implant (CI) is a well-established method of rehabilitating severe to profound hearing loss by electrical stimulation of the remaining auditory neurons, the spiral ganglion neurons (SGNs), in this way bypassing damaged or lost hair cells (Lenarz, 1998). Since SGNs are the target cells that are activated by the CI electrode, their progressive degeneration after deafening due to lack of stimulation and/or the general pathological situation leads only to limited benefit (Duan et al., 2002; Swan et al., 2008; Stöver and Lenarz, 2009; Shibata et al., 2011). The provision of drugs and neurotrophic substances may increase the survival and function of the SGNs and help in reestablishing a healthy cochlear milieu (Pfingst et al., 2015; Rask-Andersen and Liu, 2015; Schilder et al., 2019). In addition, due to the anatomic situation, the distance between the stimulating contacts of the CI electrode and the SGNs is rather large. Due to this gap, electrical signals with stronger intensity have to be used and spatial spreading of the stimulation signal occurs (Wilson, 2017). This leads to an overlap in the addressed SGNs and reduces focused neuronal stimulation which in turn leads to discriminated frequency resolution (Stöver and Lenarz, 2009; Shibata et al., 2011; Li H. et al., 2017; Yilmaz-Bayraktar et al., 2020). In a more favourable situation, the neurites resprouting from the SGNs would overcome this gap to get into close contact with the electrode in order to achieve a much more specific electrical stimulation. Current research therefore focuses on improving the survival and functionality of the SGNs and on the optimization of the electrode-nerve contact (Shibata et al., 2011; Rask-Andersen and Liu, 2015; Wilson and Dorman, 2018).

Different growth factors like the brain-derived neurotrophic factor (BDNF), the glial cell line-derived neurotrophic factor (GDNF) or the fibroblast growth factors (FGFs) were shown to be involved in the maintenance and development of SGNs in the cochlea (Duan et al., 2002; Swan et al., 2008; Stöver and Lenarz, 2009; Shibata et al., 2011; Nacher-Soler et al., 2019). From rodent experiments it is known that a lack of BNDF signaling leads to progressive hearing loss (Singer et al., 2014) and that reduced levels of BDNF in the cochleae are observed in mice with presbyacusis (Rüttiger et al., 2007). Although BDNF is crucial for maintenance of the synapses in the apical cochlear turn processing high-frequency sounds, cochlear BDNF seems to be a candidate trigger for the afferent neurodegeneration following noise trauma resulting in reduced function of the auditory nerve, thus also exerting opposing effects as shown in conditional BDNF knock-out mice (Zuccotti et al., 2012). Furthermore, BDNF is provided by hair cells in the developing cochlea, but a switch of BDNF expression from inner hair cells to supporting cells and SGNs occurs when efferent projections are reorganized from axosomatic to axodendritic (Singer et al., 2014). Thus, a long-term provision of physiological levels of BDNF might serve as a promising measure to be used in cochlear implantation (Barde et al., 1982; Kranz et al., 2014).

Together with the nerve growth factor (NGF) and the neurotrophins NT-3/4/5, BDNF belongs to the group of neurotrophins. As homodimers they all have similar structures that consist out of ≈120 amino acids (Miller et al., 1997; O’Leary and Hughes, 2003; Laske and Eschweiler, 2006). Among them, NT-3 and BDNF are important for the auditory system (Apfel et al., 1996). For example, it was shown that a sustained delivery of BDNF into the inner ear of guinea pigs, also combined with electrical stimulation derived from the CI electrode, leads to a higher survival rate of SGNs (Shepherd et al., 2008; Tan et al., 2012). Furthermore, a release of BDNF resulted in the regrowth of peripheral auditory nerve fibers in the deafened adult guinea pig ear (Budenz et al., 2015).

There are several challenges associated when considering the delivery of BDNF to the inner ear. For example, systemic therapies are severely limited by the restricted blood flow to the inner ear and poor penetration of the blood-cochlea barrier (Li L. et al., 2017). Attempts to deliver BDNF with the help of a mini osmotic pump (Gillespie et al., 2003; Gillespie and Shepherd, 2005; Wise et al., 2005; Shepherd et al., 2008) are restricted since their reservoir has to be refilled periodically as well as due to an increased risk for infections of the inner ear (Konerding et al., 2017). Although gene- and cell-based BDNF delivery was successful to induce a higher survival rate of the SGNs *in vitro* (Staecker et al., 1998; Rejali et al., 2007; Warnecke et al., 2012; Scheper et al., 2019), cell toxicity and possibly uncontrollable expression of neurotrophic factors have to be taken into account (Géral et al., 2013). Implant-associated drug delivery systems might present a more effective and safer alternative. First experimental approaches to physically (e.g., by modification of the topography) (Schlie-Wolter et al., 2013; Cai et al., 2016; Leigh et al., 2017) or chemically functionalizing the surface of the CI electrode (Ryan et al., 2006; Stöver and Lenarz, 2009) in order to enable local drug delivery off the surface have already been reported (Schmidt et al., 2018; Young et al., 2018). Apart from hydrogels (Endo et al., 2005), microspheres (Mohtaram et al., 2013), and nanoparticles (Tan et al., 2012; Wang et al., 2014; Wise et al., 2016; Li H. et al., 2017; Schmidt et al., 2018) have been established as suitable carriers for neurotrophic agents, although not all of them have been successfully attached to the implant surface (Li H. et al., 2017).

In order to improve the anatomical position of the implant electrode, i.e., to get it closer to the SGNs, several design concepts of the electrode are being tested. Pre-curved and modiolar hugging electrodes have been developed (Dhanasingh and Jolly, 2017), and experimental approaches with actuating hydrogels bending the electrode towards the modiolus have been described (Stieghorst et al., 2016). Even with these improvements, there is no direct contact between the nerves and the stimulating contacts possible. For this purpose, neurite growth from the SGNs would have to be stimulated; as the outgrowing neurites will not find their way to the electrode autonomously, a nerve guidance scaffold may be necessary to direct them to the electrode contacts. Despite the fact that the development of neuronal guidance conduits for peripheral nerves has made considerable progress (Houshyar et al., 2019), and that there is strong research on regenerative scaffolds for the treatment of spinal cord injury, only limited research has been carried out with regard to scaffolds guiding resprouting neurites of SGNs to the implant. Decellularized equine carotid arteries showed promising effects on neurite regeneration (Yilmaz-Bayraktar et al., 2020). Calcium phosphate hollow nanospheres coated on a CI and delivering GDNF were proposed to help close the anatomical gap between auditory neurons and electrodes (Li H. et al., 2017). Topographical modification of the contacts was described to allow for good attachment of ganglion neurites (Cai et al., 2016), and preferred surface patterns for neurite growth were described (Leigh et al., 2017). However, no support for the traversion through the fluid-filled lumen of the scala tympani was offered in these approaches. For this purpose, a BDNF-releasing hydrogel offering a favourable surrounding for the continued growth of neurites in the scala tympani was employed, however, without effecting direct guidance of the neurites towards the electrode contacts (Senn et al., 2017).

**FIGURE 1:**
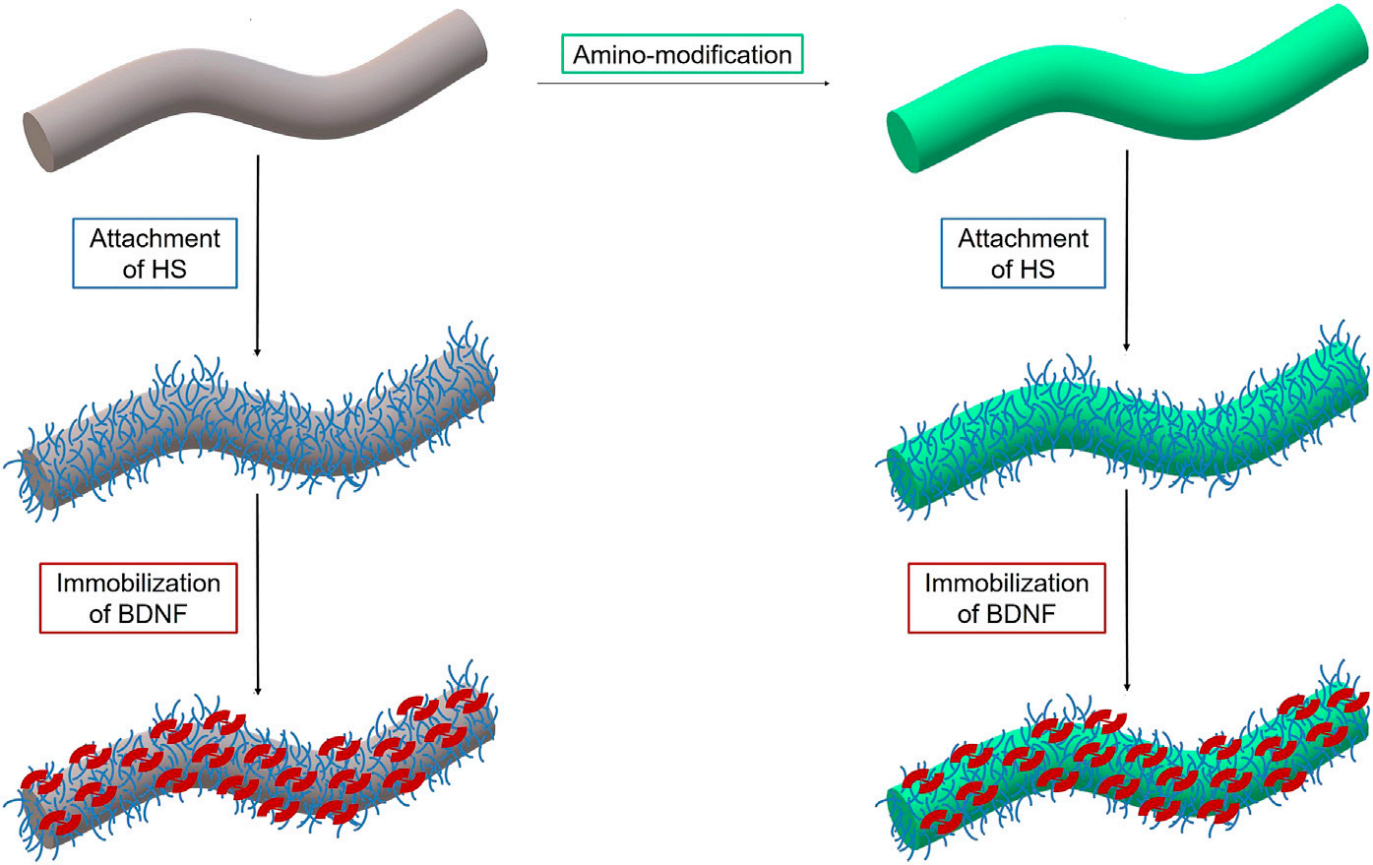
Scheme of surface functionalization of PGA/PCL polymer fiber (grey) including amino-modification (green) of the surface and subsequent attachment of heparan sulfate (blue) and loading of the growth factor BDNF (red).

We propose modified biodegradable polymer fibers as a promising neuronal guidance scaffold to guide resprouting neurites towards the surface of the CI. Here, we present how such fibers can be coated with an extra-cellular matrix component (heparan sulfate) and how this in turn can be employed to deliver a neurotrophic growth factor for an extended time (see Figure 1). The fibers have been employed either as obtained or after a chemical amino-modification step.

Whereas a nanofibrous scaffold has recently been proposed as a promising strategy for auditory nerve regeneration (Hackelberg et al., 2017), the fibers used in the present study possess diameters in the 100 μm range in order to serve as a stable support for neurites. In fact, we repurposed a long-term biodegradable suture material consisting of poly-ε-caprolactone (PCL) and polyglycolide (PGA), both polymers having been in the focus of biomedical research during the last decades (Freed et al., 1994; Woodruff and Hutmacher, 2008). The fibers were either used in their pristine form or in a version carrying amine groups. Besides their usage as absorbable suture material (Frazza and Schmitt, 1971; Bezwada et al., 1995; Middleton and Tipton, 2000), such fibers have already served as drug delivery systems for the release of growth factors in wound healing or were applied in the field of tissue engineering as an artificial extracellular matrix (ECM) (Choi et al., 2008; Yoo et al., 2009; Szentivanyi et al., 2011). The drugs were either already incorporated into the fibers during their fabrication process (Szentivanyi et al., 2011) or physically/chemically attached afterwards (Choi et al., 2008; Yoo et al., 2009).

In order to cover the surface of the fibers and mimic a natural environment, the fibers were first coated with heparan sulfate, a glycosaminoglycan of the ECM. In general, HS is known to improve wound-healing, but it is also known as being anticoagulative and analgesic. It was utilized to support survival of and neurite outgrowth from SGNs (Schulze et al., 2018). Previous studies demonstrated the enhanced biocompatibility and tissue reaction after implantation of collagen-HS-matrices in rats (Pieper et al., 2000a; Pieper et al., 2000b). In biomedical applications, HS can act as a regenerative factor to support the formation of new tissue by building up the structure of the ECM (Rabenstein, 2002; Häcker et al., 2005; Cacicol Augentropfen, 2019). It is well known that HS can bind growth factors, stabilizing (Okolicsanyi et al., 2014) and protecting them from denaturation and proteolytic degradation (Gospodarowicz and Cheng, 1986; Sommer and Rifkin, 1989; Ruoslahti and Yamaguchi, 1991). In the ECM, HS proteoglycans serve as reservoirs for growth factors and enhance their long term bioavailability (Dinbergs et al., 1996). Furthermore, growth factor-mediated signal transduction processes are activated by HS (Schlessinger et al., 1995; Chuang et al., 2010). As shown for basic fibroblast growth factor, HS proteoglycans support the internalization process of growth factors and could control the delivery process by generating stabilizing HS/growth factor complexes (Roghani and Moscatellis, 1992; Pieper et al., 2002). Also it was shown that BDNF can be delivered from glycosaminoglycan surfaces in a biologically active form over a longer period of time (Sakiyama-Elbert and Hubbell, 2000). Additionally, simultaneous treatment of SGN explants with HS and BDNF promotes enhanced neurite outgrowth (Schulze et al., 2018). Thus, HS is an appropriate matrix for the binding, modulation and sustained release of biologically active BDNF and could be used for applications in the inner ear. Correspondingly, we loaded BDNF on the HS-coated suture fibers. We studied the release of BDNF from the fibers over a period of 110 days and verified its biological activity in *in vitro* experiments with spiral ganglion cells.

## Experimental Section

### Synthetic Procedures

**FIGURE 2:**
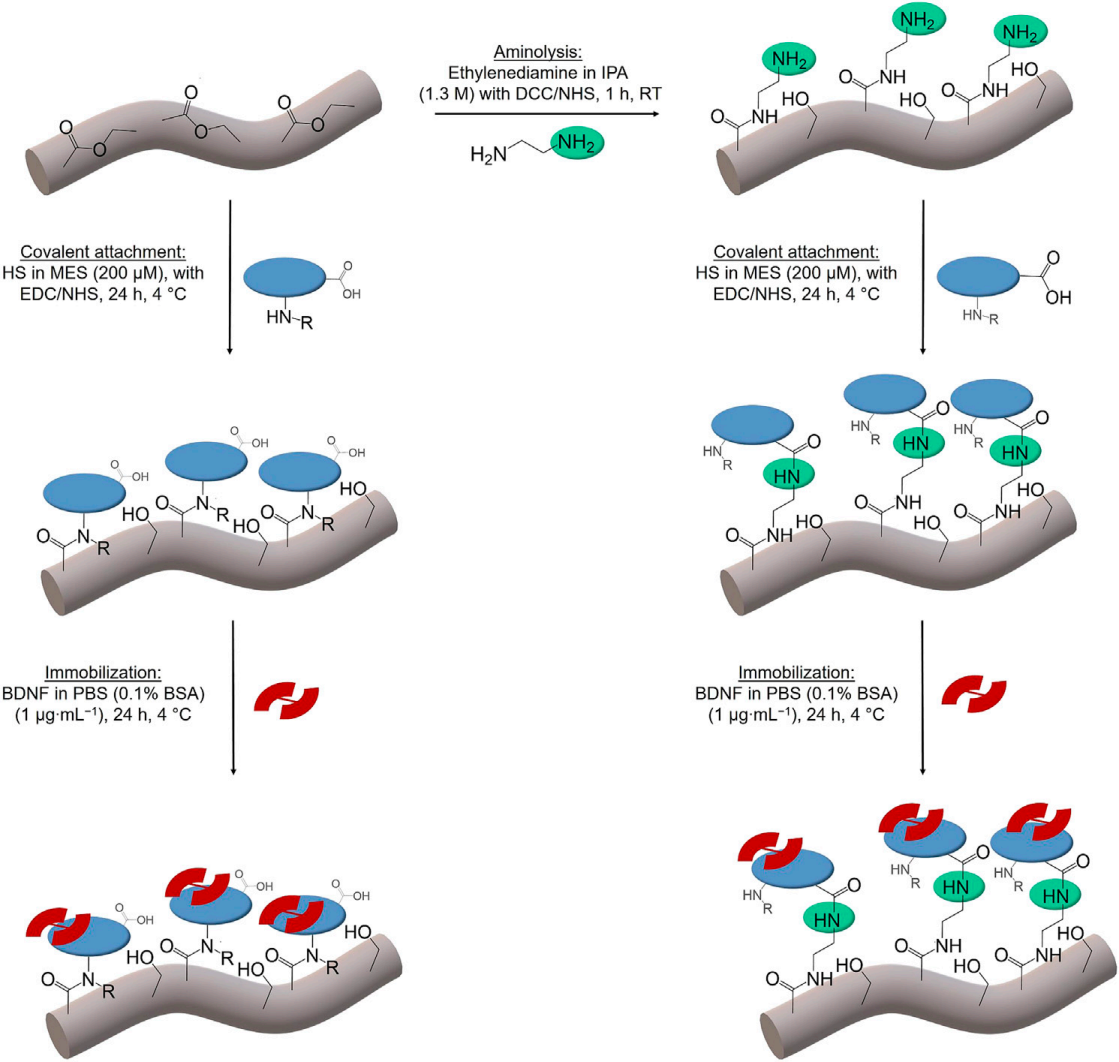
Scheme of the chemical operations used in surface functionalization of PGA/PCL polymer fibers (grey). Aminolysis with ethylenediamine (green); covalent attachment of HS (blue). Different functional groups of HS (amino (left)/carboxy (right)) lead to the coupling via amide bonds. The immobilization of BDNF (red) follows in both cases. IPA stands for isopropanol, DCC for N,N′-dicyclohexylcarbodiimide, NHS for N-hydroxysuccinimide, HS for heparan sulfate, MES for (2-N-morpholino)ethanesulfonic acid, EDC for 1-ethyl-3-(3-dimethylaminopropyl)carbodiimide, BDNF for brain derived neurotrophic factor, PBS for phosphate buffered saline and BSA for bovine serum albumin.

The scheme in Figure 2 shows the different chemical operations applied including the surface modification of the polymer fibers with ethylenediamine and their decoration with the biomolecules HS and BDNF. All chemicals were obtained commercially from Sigma-Aldrich (Munich, Germany), if not described otherwise, and were used without further purification.

#### Amino Modification of Polymer Fibers

Polymer fibers Glycolon^®^, made of polyglycolide and polycaprolactone (PGA/PCL), were purchased from Resorba (Nuremberg, Germany). Fibers were cut into pieces of 3 cm length; each sample consisted of 5 fibers. To generate free amino groups on the surface of the fibers, they were subjected to aminolysis by reacting them in a solution containing ethylenediamine (Zhu et al., 2002; Horne et al., 2010; Gloria et al., 2012). To activate the ester groups on the surface of the fibers and to later on covalently bind ethylenediamine, the fibers were firstly reacted with the coupling and stabilizing agents *N,N′*-dicyclohexylcarbodiimide (DCC) and *N*-hydroxysuccinimide (NHS). For this purpose, each sample was immersed in 2 ml of a dichloromethane (DCM) solution containing 16.5 mg DCC and 2.30 mg NHS in a 2 ml Eppendorf tube and then stirred at room temperature (r.t.) for 10 min. After washing the samples with 2 ml of DCM and 2 ml of isopropyl alcohol (IPA), each for 2 min, they were covered with a 1.3 M ethylenediamine solution (173.8 μl) in IPA (1.826 ml) and stirred for 1 h at r.t. All reactions took place in a Thermomixer from Bioer (Biozym Scientific, Hessisch Oldendorf, Germany) and were stirred at 1,500 rpm. Finally, the fibers were washed for 10 min with pure IPA, for 2 min with ultrapure water and then dried under vacuum.

#### Covalent Immobilization and Stability of Attached Heparan Sulfate

The immobilization of heparan sulfate (HS sodium salt from bovine kidney) took place in sterile solutions containing 200 μmol ml^−1^ of HS in (2-*N*-morpholino)ethanesulfonic acid-buffer (MES buffer) (50 mM, 40% (v/v) ethanol/water, pH 5.5) (Pieper et al., 2000b; Schmidt and Thull, 2003). For covalent attachment of HS, the coupling agent 1-ethyl-3-(3-dimethylaminopropyl)carbodiimide (EDC) (18 mM) and the stabilizing agent NHS (3.6 mM) were added. To later on test the stability of the attached HS, also fluorescence-labelled heparan sulfate was used in the experiments (fluoresceinamine-labelled HS from pig kidney, *λ*_ex_ = 495 nm and *λ*_em_ = 518 nm, purchased from Cosmo Bio Co., LTD.; a HS concentration of 158 μmol ml^−1^ was used in these experiments due to high costs of fluorescence-labelled HS). In order to avoid interference of the fluorescence from the labelled HS with components of the buffered solution, pure water was used as a solvent instead of MES for those experiments.

First, a sample of fibers, unmodified or amino-modified, was sterilized by placing it under UV light for 2 h. It was then incubated for 30 min in 0.5 ml of pure MES buffer or water, respectively, at 4°C in the Thermomixer under constant shaking at 1,500 rpm. Afterwards the solvent was removed and the samples were treated in 0.5 ml of the reaction solution for 24 h in the Thermomixer under the same conditions. The fibers were then washed with 2 ml of pure MES buffer or water, respectively, for 10 min, followed by 2 min washing steps first in a 0.1 M disodium phosphate (Na_2_HPO_4_) solution, then in a 1 M sodium chloride (NaCl) solution, and finally in ultrapure water; all these operations were carried out in the Thermomixer. As control experiments, one sample of unmodified and one of amino-modified fibers were treated under similar conditions, but without adding HS.

Subsequently the stability of the attached HS was tested by carrying out release experiments with the pretreated fibers. The release of HS was started by giving 1 ml PBS (0.1% BSA) to the samples. PBS is an often used standard release medium to simulate physiological conditions and has a pH value of 7.4 similar to the cochlear fluid (El Kechai et al., 2015). Mimicking the body temperature, the samples were kept at 37°C. At several time intervals, the supernatants were removed and 1 ml fresh PBS (0.1% BSA) was added to continue the release. In case of the fibers covered with fluorescent HS, all supernatants were frozen to be later analyzed by fluorescence spectroscopy. Directly after incubation as well as after 21 days release, the fibers treated with non-fluorescent HS were analyzed by zeta potential measurements.

#### Immobilization and Release of BDNF

Recombinant BDNF was purchased from Life Technologies (Darmstadt, Germany). It had been produced in *Escherichia coli* and had a purity higher than 98%. Following Horne et al. (2010), BDNF immobilization took place in sterile solutions with a concentration of 1 µg BDNF ml^−1^ in phosphate-buffered saline (PBS). The solution contained 0.1% bovine serum albumin (BSA), which acted as stabilizer of BDNF and as a filler protein to prevent BDNF adhesion to reaction tubes (Li et al., 2010).

One sample of fibers, unmodified or amino-modified and pretreated with HS, was incubated in 1 ml of the sterile protein solution for 24 h at 4°C in the Thermomixer under constant shaking of 1,500 rpm. Afterwards the samples were washed once with PBS (0.1% BSA). All incubation and washing solutions were kept frozen at −20°C to detect any leakage of protein from the fibers for later analysis by an enzyme linked immunosorbent assay (ELISA). For control conditions one sample of unmodified and amino-modified fibers was treated under similar conditions, but without BDNF additive. In order to test the ability of HS to bind and release BDNF, fibers were also treated only with the growth factor, without prior HS treatment.

Subsequently, the release of BDNF was started in a way similar to the experiments carried out with the fibers covered only with HS (see above). Similar to all supernatants from the release study, also the incubation and washing solutions were frozen to be later analyzed by ELISA. Moreover, the supernatants were subsequently used for cell culture investigations with spiral ganglion cells.

### Characterization

#### Experimental Techniques

For the imaging of the fibers, scanning electron microscopy (SEM) was performed. SEM images were taken on a JSM-6610LV from Jeol (Freising, Germany) operated with 2 kV. Attenuated total reflectance Fourier transform infrared (ATR-FTIR) spectroscopy characterization of the fibers was performed with the Fourier transformation IR spectrometer *Tensor 27* from Bruker Optik GmbH (Ettlingen, Germany). The spectra were recorded in a range of 400 cm^−1^ up to 4,000 cm^−1^. Fluorescence spectroscopy measurements were carried out using the plate reader *Spark 10* from Tecan. Zeta potential titration curves of differently modified fibers were measured with the SurPass™ 3 from Anton Paar (Graz, Austria) using a gap measuring cell with two stamps (20 × 10 mm). For each measurement, an area of 10 × 10 mm^2^ of one stamp was covered with the fibers by attaching them to double-sided adhesive tape. The fibers were stacked parallel to each other and vertically to the flow direction of the electrolyte (KCl, 0.01 M). The second stamp was covered with a glass slide (10 × 10 mm). After adjusting the pH value with aqueous KOH (0.05 M), the curves were recorded by proceeding from basic to acidic pH values by addition of aqueous HCl (0.05 M). All chemicals had analytical grade and were dissolved in ultrapure water. At each pH value, the zeta potential was measured three times with the SurPass™ 3.

#### Qualitative Determination of the Amino Groups

Fluorescein isothiocyanate (FITC, *λ*_ex_: 490 nm, *λ*_em_: 525 nm) was used to label the amino groups on the aminolyzed fibers for fluorescence measurements by immersing one sample of fibers in 2 ml of a 1 mM FITC ethanol solution for 24 h at r.t. under exclusion of light in the Thermomixer at 1,500 rpm (Lin et al., 2005). Afterwards, the fibers were washed twice with ethanol and once with ultrapure water and dried under vacuum. Fluorescence measurements were performed with the incident light fluorescence microscope BX51 from Olympus (Hamburg, Germany) with appropriate filter oculars.

#### Qualitative Determination of Heparan Sulfate

The attachment of HS to the unmodified or amino-modified fibers was assessed using toluidine blue (TB). Heparan sulfate contains esterified sulfuric acid groups and gives a metachromatic reaction with toluidine blue dye (Macintosh, 1941). Immediately after treatment with HS, the samples were covered with 1 ml of toluidine blue solution (18.5 µM, 10 mM HCl, 0.2% NaCl) and treated for 30 s in an ultrasonic bath to form the complex. For the removal of any HS-dye complex that got into the solution, extraction with hexane followed. 1 ml of hexane was added and the samples were treated for 30 s in an ultrasonic bath. The absorption intensity of the remaining TB solution was determined afterwards. Absorption measurements took place at 631 nm on an EON spectrophotometer (Biotek, Winooski, United States).

#### ELISA

The amount of immobilized and released BDNF was quantified by an enzyme-linked immunosorbent assay (ELISA) kit against human BDNF (Boster Biological Technology Co., Ltd., Pleasanton, United States). The BDNF-ELISA kit was used in accordance to the manufacturer’s recommendations. A brief summary of the procedure follows. The 96-well plate delivered had been pre-coated with a monoclonal antibody for BDNF. Standards and samples were diluted in sample dilution buffer and added to the wells for 90 min. After the incubation, the plate content was discarded and a working solution of a biotinylated anti-human BDNF antibody was added for 60 min. After three washing steps with PBS, an incubation with a working solution containing an avidin-biotin-peroxidase complex followed (30 min). Further washing steps with PBS to remove the unbound complex were performed. For colorimetric detection 3,3′,5,5′-tetramethylbenzidine was added. The color development was stopped with 2 M sulfuric acid. All incubation steps were performed at 37°C. Absorbance was recorded at 450 nm on an EON spectrophotometer (Biotek, Winooski, United States).

### Cell Culture Investigations

#### NIH 3T3 Fibroblasts

According to Williams et al. (2015) the initial cytocompatibility tests were performed with the murine fibroblast cell line NIH 3T3 (ATCC-Number: CRL-165). The unmodified and amino-modified fibers were sterilized under UV light. NIH 3T3 fibroblasts were first cultivated in high glucose Dulbecco’s Modified Eagle’s Medium (DMEM, Biochrom, Berlin, Germany) with supplements of 10% fetal calf serum (FCS, Biochrom, Berlin, Germany), 1% penicillin and streptomycin (Biochrom, Berlin, Germany). Cells were seeded with a density of 1 × 10^4^ cells per well in a 48-well plate (TPP, Trasadingen, Switzerland) and then the cultivation took place for 3 days at 37°C in a humidified atmosphere (5% CO_2_) for expansion. After removing the medium, 100 µl fresh medium and one unmodified or amino-modified fiber was added to one well. Measurements in each condition took place in triplicates. Incubation was performed for 4 days and every day the morphology and proliferation of the fibroblasts was checked with a transmission light microscope (CKX41, Olympus, Hamburg, Germany) with a CCD-camera (Colorview III, SIS, Olympus).

#### Neutral Red Uptake Assay

The cell viability of the NIH 3T3 fibroblasts was determined by the neutral red uptake (NRU) assay after the incubation. First, neutral red (3-amino-7-dimethylamino-2-methyl-phenazine hydrochloride, Merck, Darmstadt, Germany) stock solution was prepared by dissolving 40 mg neutral red dye in 10 ml purified water (4 mg ml^−1^). Then the stock solution was diluted 1:50 in pre-heated (37°C) DMEM. After removing the medium from the well, 100 µl of fresh neutral red-containing medium were added per well. An incubation of 3 h at 37°C and 5% CO_2_ followed and the neutral red medium was discarded. Afterwards, the cells were washed and fixed by adding solution I (1% calcium chloride, 0.5% formaldehyde in purified water) for 5 min incubation time. After removal of this solution, 100 µl of a neutral red destaining solution (1% acetic acid, 50% ethanol (95%) in purified water) were added. The plate was shaken and incubated for 10 min at 4°C. The absorption of the neutral red extract was measured at 570 nm using a microplate reader (Synergy™ H1, BioTek, Bad Friedrichshall, Germany).

#### Ethics Statement for Isolation of SGNs From Neonatal Rats

The experiments and analysis of this study were conducted from November 2016 to May 2019. All experiments were carried out in accordance with the European Directive 2010/63/EU for the protection of animals used for experimental purposes and with the institutional guidelines for animal welfare of the Hannover Medical School following the standards described by the German “Law on Protecting Animals” (Tierschutzgesetz). Animals were euthanized to yield the tissue for our *in vitro* experiments. This procedure is registered (no.: 2016/118) with the local authorities (Zentrales Tierlaboratorium, Laboratory Animal Science, Hannover Medical School, including an institutional animal care and use committee) and reported on a regular basis as demanded by law. For exclusive sacrifice of animals for tissue analysis in research, no further approval is needed if no other treatment is applied beforehand (§4). All rats were bred and born for research study purposes. A breeding stock was supplied by Charles River (Charles River, Wilmington, United States) and housed with their litters in the facilities of the licensed Institution of Laboratory Animal Science of the Hannover Medical School. Neonatal rats were rapidly decapitated prior to any experimentation by a licensed person to minimize their stress level.

#### Spiral Ganglion Cell Culture

The neuroprotective effects of BDNF released from unmodified and amino-modified fibers were investigated in cell culture investigations with spiral ganglion cells (SGCs). Cells were obtained by dissociation of spiral ganglia resulting in mixed cell cultures containing neurons, fibroblasts and glial cells. The spiral ganglia were isolated from neonatal Sprague-Dawley rats (postnatal day 3–5) that were sacrificed by rapid decapitation. The dissection of the cochleae and the mechanical and enzymatic dissociation of the spiral ganglia were performed according to a previously described protocol (Wefstaedt et al., 2005; Warnecke et al., 2012). With the help of a Neubauer chamber (Brand, Wertheim, Germany) and the trypan blue staining (Sigma Aldrich, Munich, Germany), the number of viable cells was determined. Afterwards, the dissociated cells were seeded at a density of 1 × 10^4^ cells per well in a 96-well plate (TPP). The used plates were coated with poly-*D*/*L*-ornithine (0.1 mg ml^−1^) and laminin (0.01 mg ml^−1^, Life Technologies, Carlsbad, United States) prior to cell seeding. Equal volumes of the released supernatants (50 µl) were added to the wells containing the SGNs (in 50 µl medium). In addition to the tested supernatants, experiments always included a negative control (SGCs cultivated in medium), a positive control (SGCs in medium with 50 ng ml^−1^ BDNF additive), and an additional control of the solution of the supernatants (medium with PBS and 0.1% BSA) (1:1). Until they were added to the SGC culture, the supernatants had been frozen. The incubation was performed in serum-free medium (Panserin 401, PAN Biotech, Aidenbach, Germany), which was supplemented with HEPES (25 mM, Life Technologies, Carlsbad, United States), glucose (6 mg ml^−1^, Braun AG, Melsungen, Germany), penicillin (30 U ml^−1^, Grünenthal GmbH, Aachen, Germany), N-2 supplement (3 μg ml^−1^, Life Technologies, Carlsbad, United States) and insulin (5 μg ml^−1^). After 48 h of cultivation at 37°C and 5% CO_2_, the cells were fixed with a 1:1 methanol (Carl Roth, Karlsruhe, Germany) and acetone (J.T. Baker, Arnhem, Netherlands) solution for 10 min and washed with PBS (Gibco^®^ by Life Technologies, Carlsbad, United States). A seeding control was fixed already 4 h after the seeding of SGNs.

#### Evaluation of the Survival Rate of SGNs

As the cultures from dissociated spiral ganglion cells consisted of a mixture of neurons, fibroblasts and glial cells, the SGNs had to be identified by staining. For this purpose, a monoclonal mouse 200 kDa-neurofilament antibody (clone RT97, Leica Biosystems, Wetzlar, Germany) was used as a neuron-specific marker. Briefly, fixed cells were first incubated with the primary neurofilament antibody, then washed with PBS and incubated with a secondary biotinylated anti-mouse antibody (Vector Laboratories Inc., Burlingame, United States). Afterwards, they were reacted with an ABC complex solution (Vectastain Elite ABC-Kit, Vector Laboratories Inc., Burlingame, United States) according to the manufacturer’s protocol (Wefstaedt et al., 2005), to be then visualized with diaminobenzidine (Peroxidase Substrate Kit DAB, Vector Laboratories Inc., Burlingame, United States). Imaging of cells was performed with an inverted microscope (CKX41, Olympus, Hamburg, Germany) to determine the number of survived SGNs. These were defined as neurofilament-positive cells with a neurite length of at least 3 cell soma diameters (Gillespie et al., 2001). By relating the number of survived neurons per well to the seeding density of the control sample in the same plate after 4 h cultivation, the survival rate was calculated.

#### Evaluation of the Neurite Length of SGNs

Furthermore, the neuroregenerative effect of the supernatants was examined by imaging the five longest neurons in each field of view (one in the center and four around the perimeter of the well) with an inverted microscope (Olympus CKX41) including a CCD-camera (Colorview III, SIS, Olympus). To measure the length of each neuron, the imaging software CellP (SIS) was used. In this procedure, all conditions were blinded for the analysts.

#### Statistical Analysis

All statistical analyses were performed with Prism 5 (GraphPad, La Jolla, United States). For validation of the results, a one-way analysis of variance (ANOVA) followed by Tukey’s Multiple comparison test was used; *p* values of less than 0.05 were considered to be statistically significant. All quantitative data represent the means of at least two independent approaches (*N*), including at least triplicates of each sample (*n*). Error bars in the figures indicate the standard error of the mean. Levels of significance are indicated as follows: n.s. = not significant, *p < 0.05; **p < 0.01; ***p < 0.001.

## Results and Discussion

### Characterization of the Polymer Fibers

Two different types of fibers were chosen as the basis for all investigations within this paper. We used polymer fibers Glycolon^®^ from Resorba, which are made of polyglycolide and polycaprolactone (PGA/PCL). In order to activate the surface of these fibers for further chemical reactions, the fibers were subjected to an aminolysis. For this purpose, the fibers were incubated in an ethylenediamine solution in isopropanol for 1 h after they had been treated with the coupling agent DCC and the stabilizing agent NHS in order to covalently bind the diamine to the surface of the fibers. The two types of fibers are designated as “unmodified” and “amino-modified” in the following.

**FIGURE 3:**
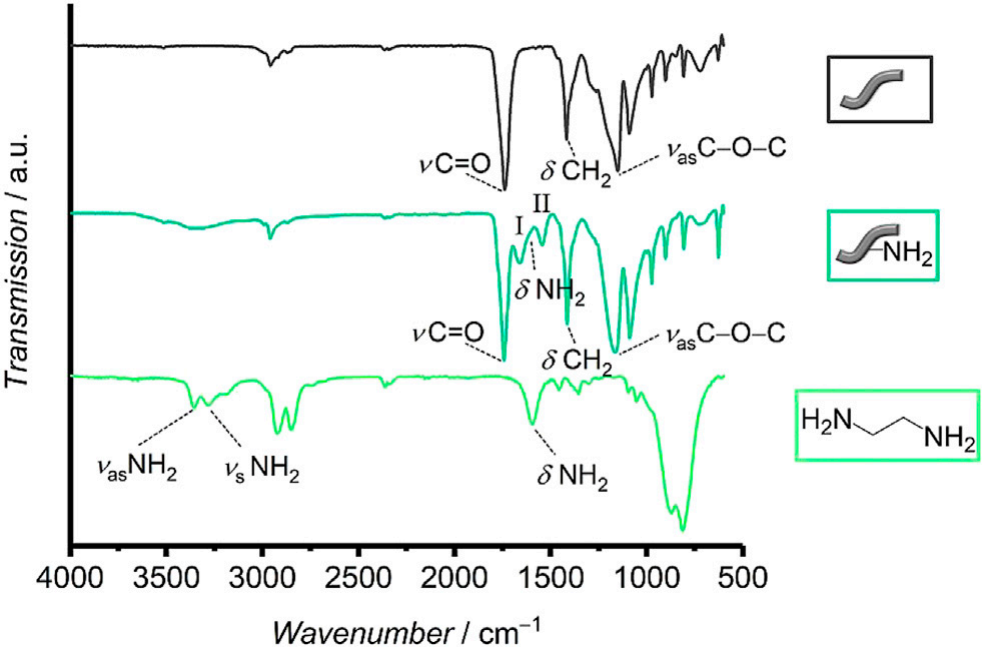
Infrared spectra of unmodified and aminolyzed fibers as well as of ethylenediamine. In the spectrum of the aminolyzed fibers the bands of the two amide vibrations I and II are marked.

For overall characterization of the two types of fibers, ATR-FTIR spectroscopy measurements were performed (Figure 3). The spectra of both types of fibers show typical vibrational bands for polyesters like poly-ε-caprolactone and polyglycolide (Günzler and Gremlich, 2003; Hesse et al., 2005; Spearman et al., 2014). Representatively, an intense C=O stretching vibration appears at 1739 cm^−1^ for the unmodified and at 1743 cm^−1^ for the aminolyzed fibers, respectively. Furthermore, both spectra show a CH_2_ deformation vibration (at 1,417 cm^−1^ for unmodified and at 1,413 cm^−1^ for aminolyzed fibers, respectively) as well as bands for an asymmetric C−O−C stretching (at 1,151 cm^−1^ for unmodified and at 1,163 cm^−1^ for aminolyzed fibers, respectively) and a symmetric C−O−C stretching (at 1,091 cm^−1^ for unmodified and at 1,089 cm^−1^ for aminolyzed fibers, respectively). The signal at 974 cm^−1^ can be attributed to an asymmetric C−O−C deformation vibration of the ester group for both types of fibers. Furthermore, a C−H deformation vibration appears at 902 cm^−1^, also for the two types of fibers. These results do not only show that the samples are made out of hydrocarbons with ester groups, as expected, but they also confirm that the bulk chemical composition of the fibers has not changed significantly after treatment with ethylenediamine. Nevertheless, some additional vibrations appear in the spectrum of the aminolyzed fibers that prove the successful amino-modification. A broad IR band of low intensity in the range from 3,700–3,100 cm^−1^ in the spectrum of the aminolyzed fibers can be related to symmetric and asymmetric NH_2_ stretching vibrations. Moreover, the spectrum of the aminolyzed fibers additionally shows a strong band at 1,660 cm^−1^, corresponding to C=O stretching vibrations of amide groups (amide I band). A slight shoulder towards smaller wavenumbers can be identified, which can be assigned to NH_2_ deformation vibrations of the primary amino groups of the ethylene diamine. The strong band at 1,545 cm^−1^ can be related to the C−N−H deformation vibration and C−H stretching vibration of the amide groups (amide II band) (Günzler and Gremlich, 2003). This verifies a successful covalent attachment of ethylenediamine residues to the fibers. As this reaction will predominantly have taken place on the surface of the fibers, this results in the presence of free amino groups on the surface.

**FIGURE 4:**
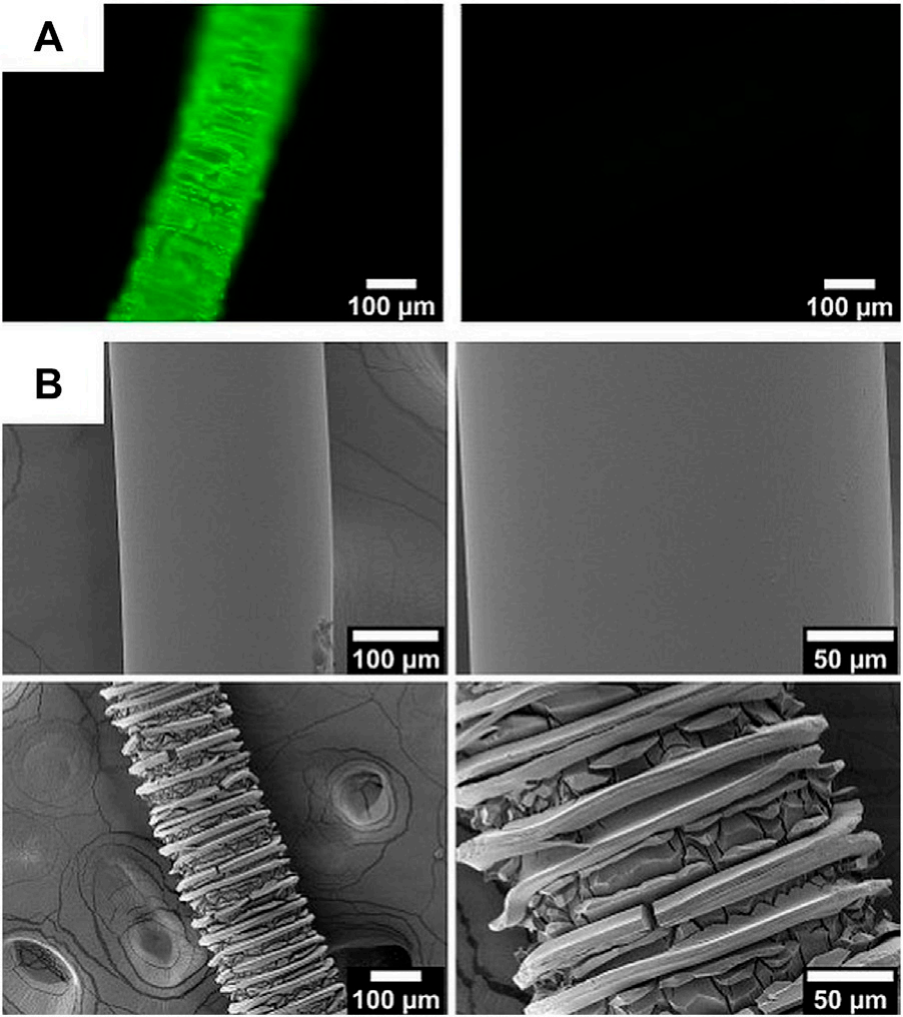
**(A)** Fluorescence microscopy images of aminolyzed (left) and control fibers (right) after treatment with FITC. **(B)** SEM images of unmodified (top) and amino-modified fibers (bottom) at two different magnifications.

In order to visualize the primary amino groups on the fibers, they were labeled with fluorescein isothyocanate (FITC) and visualized by fluorescence microscopy. The thiocyanate moieties of FITC will react with primary amino groups resulting in covalent binding of the fluorescein fluorophores on the polymer fibers that cannot be rinsed out. In contrast to this, the ester groups on the surface of the fibers cannot react with FITC under the same conditions. As can be seen in Figure 4A, the aminolyzed fibers (left) show the expected green fluorescence whereas the unmodified fibers have not bound any fluorescein (right).

The fluorescence microscopy image (Figure 4A left) of the aminolyzed fibers already indicates a rough surface of the fiber. In fact, SEM investigations of the unmodified and the amino-modified fibers (Figure 4B reveal that the surface is partly destroyed by the aminolysis treatment. This can be stated based on the decreasing diameter of the fibers (from 245.0 ± 0.6 µm down to 209 ± 14 µm) and the appearance of many cracks on the surface of the fibers. These cracks appear in a regular fashion which may in some way be related to the production process of the fibers. The reason for the partial destruction can be found in the strong basicity of the diamine solution (Zhu et al., 2002). As can be inferred from a comparison of the SEM images in Figure 4B, the amino-modified fibers thus offer an enhanced surface area which may allow the attachment of larger amounts of biomolecules; the roughness may improve cell adhesion in future applications of such aminolyzed fibers.

**FIGURE 5:**
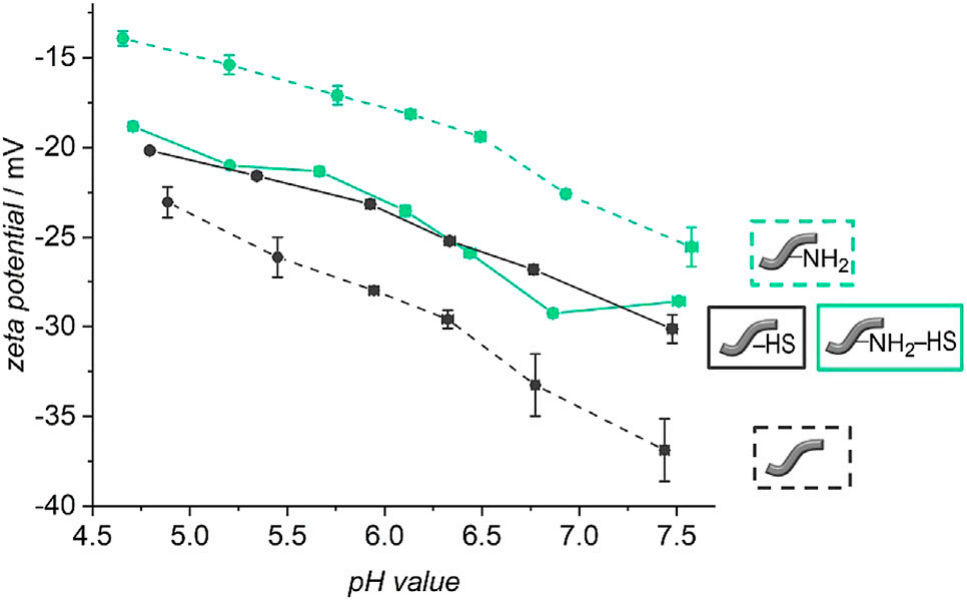
pH-dependent zeta potential titration curves of unmodified (dashed grey line) and amino-modified (dashed green line) fibers as well as for those fibers coated with heparan sulfate: unmodified fibers (continuous grey line) and amino-modified fibers (continuous green line).

Zeta potential measurements carried out under variation of the pH provide further evidence for the successful modification of the fiber surface and show directly their altered surface characteristics (see Figure 5). The curves of the unmodified and the amino-modified fibers differ strongly from each other within the physiological pH range investigated. The unmodified fibers show a strongly negative zeta potential, decreasing from −23.5 ± 0.9 mV at pH ≈ 5 to −36.9 ± 1.7 mV at pH ≈ 7.5. This is probably due to carboxylic acid groups on the surface which result from cleavage of ester groups or had not reacted during polyester formation (You et al., 2005; Bajgai et al., 2008). In contrast, the amino-modified fibers possess considerably less negative values of the pH value-dependent zeta potential. The values start at −14.0 ± 0.4 mV at the acidic pH ≈ 5 and decrease to about −25.6 ± 1.1 mV at pH ≈ 7.5. This change in zeta potential can be easily traced back to the successful aminolysis because the additional amino groups have an alkaline character and can be protonated, leading to more positively charged surfaces.

### Attachment of Heparan Sulfate

**FIGURE 6:**
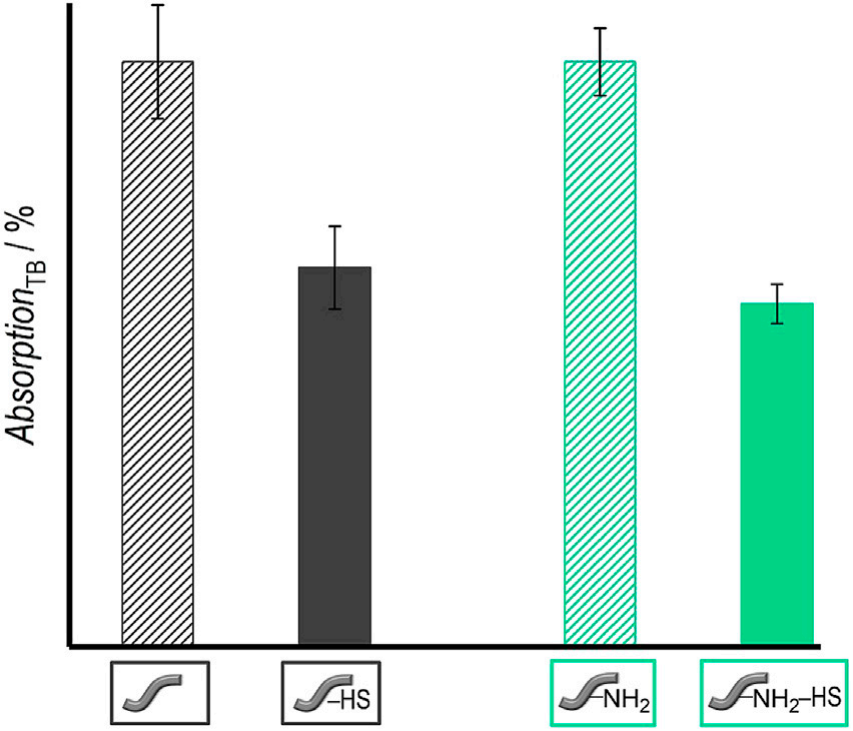
Results of TB assay: Absorption of TB solutions after contact with different types of fibers: Unmodified fibers without HS (grey stripes)/with HS (grey) as well as amino-modified fibers without HS (green stripes)/with HS (green) (right). Values are given as mean ± standard error of the mean (*N* = 3).

The attachment of heparan sulfate (HS) to the surface of the polymer fibers was carried out by incubating them in an HS solution in MES for 24 h with added EDC and NHS to covalently bind HS to the surface of the fibers. As a qualitative test, the presence of HS on the fibers was verified by using the toluidine blue (TB) assay (Macintosh, 1941; Kang et al., 1996; Rohman et al., 2009). After the incubation of the treated fibers in the TB solution, the remaining absorption of the dye in the solution is measured by UV-Vis spectroscopy at 631 nm. According to Macintosh (Macintosh, 1941), the TB dye forms a complex with the esterified sulfuric acid groups of HS; TB attached to the polysaccharide bound on the surface of the fibers is thus removed from the solution, resulting in a lower absorption of TB in the solution. Figure 6 shows that the absorption and thus also the concentration of TB molecules in the solution decreases when fibers carrying HS were present before—in contrast to the control solutions exposed to fibers without HS. Both fibers, unmodified and amino-modified, show similar results (Table 1). In the case of the unmodified fibers, the intensity of the surrounding TB solution decreases by approximately 35 ± 7% and in case of the amino-modified fibers the TB intensity decreases by 41 ± 3%. Thus, this qualitative test shows a positive result for the similar attachment of HS to both types of fibers.

**TABLE 1:** Absorption of TB solution after contact with unmodified fibers without and with HS as well as amino-modified fibers without and with HS.

	Intensity of absorption of TB solution/%	
Type of fiber	With heparan sulfate	Without heparan sulfate
Unmodified	64.8 ± 7.1	100 ± 9.7
Amino-modified	58.6 ± 2.9	100 ± 5.8

The zeta potential titration curves (see Figure 5) of the HS-decorated unmodified and amino-modified fibers supply further evidence for changes in the surface characteristics after reaction with HS. Both curves − from HS-decorated unmodified and amino-modified fibers—are very similar to each other. Independently of the initial fiber type, both materials exhibit zeta potentials ranging from about −20 mV to about −30 mV in the addressed range of pH values from 5 to 7.5. This finding can be rationalized by the assumption that the surface chemistry of both materials is essentially governed by the same substance, namely heparan sulfate, and that the actual composition of the fiber surface, non- or amino-modified, only plays a subordinate role. HS is a linear polysaccharide carrying acidic carboxy and sulfate groups. Therefore, in the physiological range of pH values considered here, HS always carries a negative charge. It has been noted that for long range interactions with proteins, this charge is important in attracting proteins to HS via charged binding sites in lysine and arginine residues (Rabenstein, 2002; Meneghetti et al., 2015). The zeta potential of free HS in PBS solution lies at −10 mV (Kalaska et al., 2019), so the polysaccharide is somewhat less strongly negatively charged than in our case of surface-bound heparan sulfate. This may be explained by the fact that the actual charge on polyelectrolytic polymers depends on the conformation of the polymer strands. In any case, the pH value-dependent zeta potential titration measurements confirm the attachment of HS to both types of fibers. This finding also indicates that the fiber surface is effectively covered by a layer of a substance which is part of the extracellular matrix.

**FIGURE 7:**
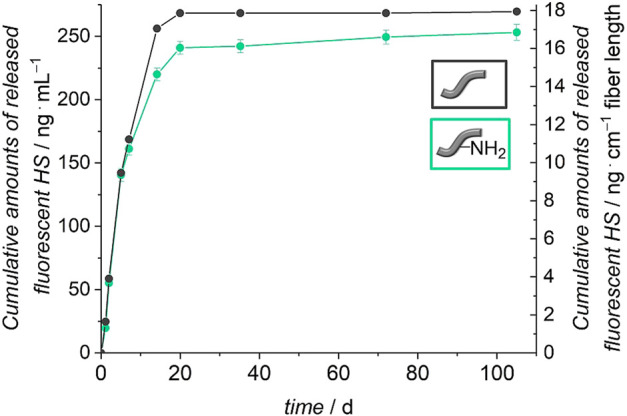
Release of HS from unmodified and amino-modified (PBS with 0.1% BSA, 110 days, 37°C). The left axis represents the cumulative HS release referred to 1 ml release medium and the right axis shows the cumulative release of HS with regard to 1 cm fiber length.

In order to find out whether HS is attached covalently to the surface of the fibers and not only physically adsorbed, it was tested if and how much HS can be removed from the fibers. Release experiments of five fibers in PBS with 0.1% BSA were carried out at 37 °C. The stability of the attachment of the HS was investigated by using fluorescence-labelled molecules. The released amount of HS was determined by fluorescence spectroscopy measurements. Figure 7 shows the cumulatively released amount of HS from the unmodified and amino-modified fibers over a period of 110 days. Both types of fibers show similar release profiles. A burst release of HS for the first 21 days is followed by a plateau until the end of the release. Both curves indicate that the release is nearly finished after 21 days. In total 270 ng ml^−1^ HS were released from the unmodified fibers and 253 ng ml^−1^ were released from the amino-modified fibers.

**FIGURE 8:**
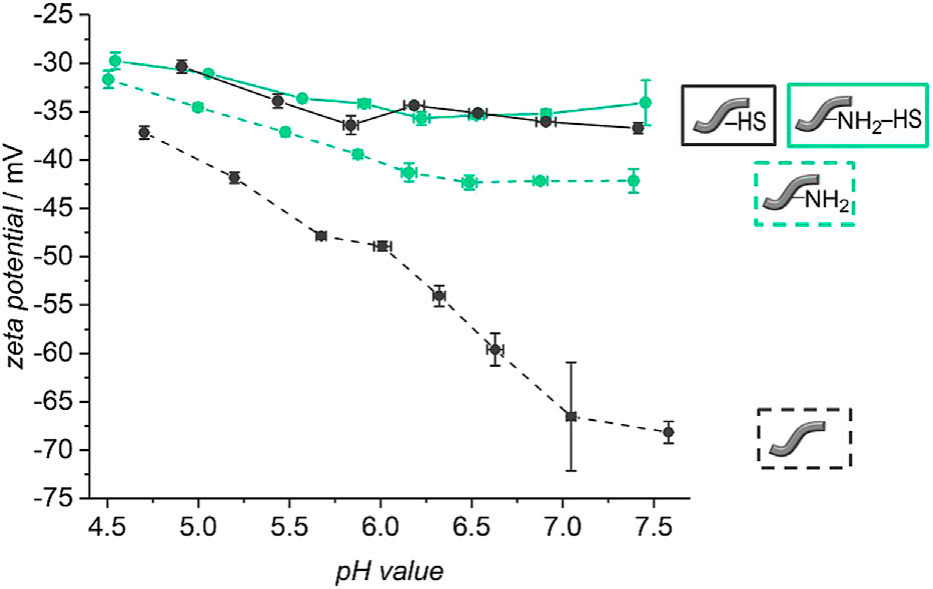
pH-dependent zeta potential titration curves of unmodified (dashed grey line) and amino-modified (dashed green line) fibers as well as for unmodified (continuous grey line) and amino-modified (continuous green line) fibers covered with heparan sulfate after a release of 21 days in PBS (with 0.1% BSA, 37°C).

Much more HS had been used in the incubation (100 μg ml^−1^) than was released from the fibers (≈260 ng ml^−1^). We wanted to find out if the release of HS in the beginning is due to weakly adsorbed molecules to the surface of the fibers and if covalently and firmly attached HS is still present on the fibers after 21 days. For this purpose, fibers treated with non-fluorescent HS were submitted to release experiments for 21 days. Afterwards, zeta potential measurements under variation of the pH were carried out with these fibers. For a better comparison, also un- and amino-modified fibers without HS were subjected to the release experiment and measured afterwards. These curves do not only show a partial degradation of the polymer fibers after 21 days of release but also a preservation of the varying surface characteristics of differently modified fibers (see Figure 8). Similar to Figure 5, the curves of the unmodified and amino-modified fibers differ strongly from each other in the investigated pH range. The unmodified fibers possess a negative zeta potential, decreasing from −40 mV at pH ≈ 5 to −68 mV at pH ≈ 7.5. Again, this is probably due to carboxylic acid groups on the surface of the fibers created by cleavage of ester groups. The fact that the zeta potential of the unmodified fibers after the release procedure is much more negative than that before the release (differences Δζ range from 16 to 31 mV), can be explained by the degradation of the surface of the fibers which occurs by hydrolysis (ester cleavage) and creates acidic carboxylic groups (You et al., 2005; Bajgai et al., 2008). In contrast, the pH value-dependent zeta potential of the amino-modified fibers after 21 days of release is much less negative when compared to the unmodified fibers. Starting at −35 mV at pH ≈ 5 the zeta potential decreases to about −42 mV at pH ≈ 7.5. This difference in zeta potential shows that the basic amino groups on the surface of the fibers are preserved; these can be protonated and thus result in a more positively charged surface. The finding that the values are more negative than those of the corresponding amino-modified fibers before the release (differences Δζ range from 17 to 21 mV) again is an indication for the degradation of the fibers. Besides the formation of carboxylic acid groups due to ester cleavage (You et al., 2005; Bajgai et al., 2008), also a release of surface-bound ethylenediamine can be considered. These findings are supported by our measurements of the pH values of the surrounding release solutions (see ESI, Supplementary Figure S1). After 21 days, the pH values observed are higher in case of the amino-modified fibers compared to the unmodified fibers in agreement with the additional release of basic substances.

Looking at the zeta potential titration curves of the HS-decorated unmodified and amino-modified fibers after the release, one can see that both curves resemble one another, similar as is the case for the fibers before the release experiments. In the physiological pH range from 5 to 7.5, the fibers after the release possess zeta potentials ranging from about −30 mV to about −35 mV. This can again be explained by the fact that, independently of the initial fiber type, the surface chemistry is dominated by the same substance, namely heparan sulfate. However, there is a difference in the absolute values, where the zeta potential of the HS-decorated fibers measured after the release experiments is only slightly more negative (differences Δζ range from 5 to 10 mV). Potentially, HS protects the fibers from strong degradation either due to sterical reasons, as HS may cover the surface of the fibers, or due to the acidic character of the glycosaminoglycan (Rabenstein, 2002; Kalaska et al., 2019). In contrast to base-catalyzed ester hydrolysis, acidic catalysis takes place more slowly and reversibly. Only in highly acidic environment (approx. pH 1), an efficient acid-catalyzed ester hydrolysis takes place (Budweiser, 2015). However, this is not the case here. All in all, our findings confirm the successful preservation of the attached HS to the surface of the polymer fibers after 21 days of release. As Figure 7 illustrates that no more HS is released from the fibers from that point-of-time on, we suggest that the remaining HS is firmly attached to the fibers, probably by covalent bonding. The small amount of HS released from the fibers during the first 21 days then corresponds to weakly adsorbed polysaccharide molecules. As the zeta potential measurements indicate, even after the release of these small amounts, plenty of HS is still present on the fiber surface, sufficient to essentially dominate the surface properties. Previous studies had already demonstrated the successful covalent attachment of glycosaminoglycans (GAGs) like HS with reactive coupling agents to PCL scaffolds (Singh et al., 2011) and to surfaces presenting amino groups (Pieper et al., 2000b; Schmidt and Thull, 2003). The immobilization of HS on the two different types of fibers can be attributed to two different types of bonds. In case of the unmodified fibers, carboxylic acid groups, generated on the surface of the fibers, are activated by the reactive coupling agents EDC/NHS to react with the amino groups of HS. Stable amide bonds then lead to a firm covalent attachment of HS (Carraway and Koshland, 1972; Hoffman et al., 1972; Ito et al., 1986; Kang et al., 1996; Singh et al., 2011). In addition, the carboxylic acid and hydroxyl groups on the fibers as well as intact ester groups can interact with corresponding groups of HS via hydrogen bonds. In case of the amino-modified fibers, the free amino groups and, probably, residual carboxy groups on the surface can be activated by the EDC/NHS system (Kang et al., 1996). Stable amide bonds can be created either between the free amino groups on the surface of the fibers and the carboxylic acid groups of the HS or between the residual carboxy groups on the fiber surface and the amino groups contained in HS. The negatively charged HS (Rabenstein, 2002) can additionally interact electrostatically with the positively charged amino groups, and, similar as for the unmodified fibers, there are numerous possibilities for hydrogen bond interactions. All in all, the number of carboxylic acid or amino groups, respectively, of the HS taking part in covalent bond formation to the fibers is small in comparison to the total number of the respective group, as the zeta potential curves for both types of fibers are very similar.

### BDNF Loading and Release

The HS-coated fibers were incubated in a solution of BDNF in PBS (0.1% BSA) for 24 h to immobilize the protein to their surface. The amount of immobilized BDNF was quantified indirectly by ELISA by measuring the remaining concentrations of the protein in the incubation and washing solutions (Table 2). As the concentrations of BDNF found in these solutions were very small (in the low ng ml^−1^ ranges vs. 1 μg ml^−1^ in the incubation solution), both types of fibers have immobilized nearly the total amount of BDNF. However, some protein molecules may have undergone conformational changes during incubation, for example by ad- and desorption to the fiber surface; these would not contribute to the ELISA signal when they are no longer immunologically active. Thus, caution is advised when interpreting the results, and immobilized amounts, which were determined indirectly, may actually be too high. Control experiments, where an incubation of BDNF took place without fibers, show the stability of the growth factor during the operations, as all BDNF can be retrieved by the ELISA.

**TABLE 2:** Quantification of the amount of immobilized BDNF on unmodified and amino-modified fibers coated with HS which were incubated in a solution containing 1 μg ml^−1^ BDNF.

Type of fiber	BDNF concentration		Apparent loading efficiency (%)
	Remaining in solution (ng ml^−1^)	Present in washing solution (ng ml^−1^)	
Unmodified−HS	5.0 ± 2.1	6.3 ± 3.2	98.9 ± 0.8
Amino-modified−HS	4.2 ± 0.9	2.5 ± 0.6	99.3 ± 0.2

BDNF acts on an extracellular receptor. Although it cannot be finally excluded that also surface-bound BDNF exerts an effect, it appears much more probable that dissolved molecules of the growth factor reach the receptors. Therefore, a covalent attachment of the BDNF on the fibers is not aimed at. Instead, we rely on a biomimetic approach, as it is well-known that one of the biological roles of HS as an extracellular matrix component is to bind and release growth factors like BDNF (Roghani and Moscatellis, 1992; Pieper et al., 2002; Okolicsanyi et al., 2014). Other interactions between the protein and the fiber surface may contribute further to the binding and release of BDNF. The fibers can additionally interact with BDNF electrostatically, as both types show a similarly negative zeta potential at a pH value of 7.4 (see Figure 5) after decoration with HS. This pH value corresponds to that of PBS, and BDNF, as a basic protein with an isoelectric point of approximately 10, has a net positive charge at this pH value (Barde et al., 1982; Mohtaram et al., 2013). Hydrogen bonds between ester, carboxyl, hydroxyl and amino groups on the surface of the polymer fibers and various moieties of the protein will add up, as will hydrophobic interactions between the nonpolar regions of the polymer surface and corresponding domains of BDNF (McDonald and Chao, 1995).

**FIGURE 9:**
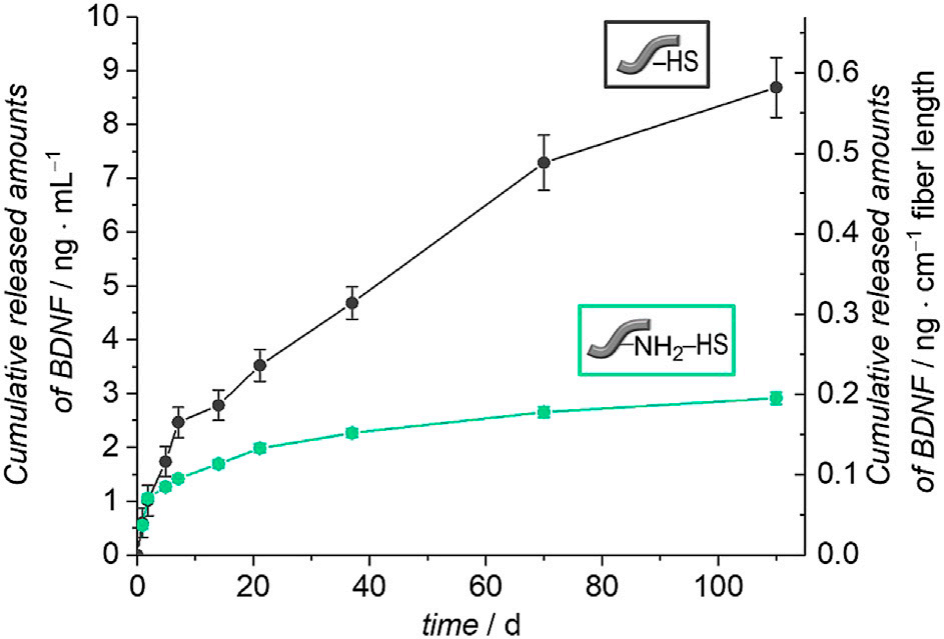
Release of BDNF from unmodified and amino-modified fibers pre-coated with heparan sulfate (HS) (PBS with 0.1% BSA, 110 days, 37°C). The left axis represents the cumulative BDNF release referred to 1 ml release medium and the right axis shows the cumulative release of BDNF with regard to 1 cm fiber length.

Investigations of the release profiles of BDNF were performed by incubating five fibers in PBS with 0.1% BSA at 37°C. The released amounts of BDNF were determined at different time points by ELISA. Figure 9 shows the cumulatively released amount of BDNF from the unmodified and amino-modified fibers, which were pre-coated with HS, over a period of 110 days.

The unmodified fibers coated with HS show a sustained release of BDNF and after 110 days, a total of 9 ng ml^−1^ had been released, which corresponds to 2% of the apparently immobilized amount of BDNF. The amino-modified fibers pre-coated with HS show a similar though less pronounced sustained release of BDNF. In total, 3 ng ml^−1^ were released in the period of 110 days, which equals 1% of the originally attached BDNF. Both curves indicate that the release is not finished after 110 days; further release is expected to be more pronounced from the unmodified fibers with HS pre-coat due to the still steeper increase of the cumulative release after long release times. In this context, it has to be noted that very high levels of BDNF, as observed for example after permanent noise-induced trauma, could also be detrimental and differential regulation of BDNF and its pro-forms as well as its receptors needs further consideration (Meltser et al., 2010).

The importance of the heparan sulfate coating becomes obvious when the release curves are compared between fibers carrying HS or not. For this purpose, in Figure 11 we depict relative release curves where the total amount released is set to 100% for each curve. Without the HS pre-coat, both types of fibers, unmodified and amino-modified, show a burst release of BDNF (dashed lines), whereas in both cases where HS is present on the fibers, a more sustained release is observed (full lines). This result is in line with our biomimetic approach where HS serves as a mimic of the extracellular matrix in storing, stabilizing and slowly releasing the growth factor. It also supports our findings of a successful and long-lasting stable attachment of HS to the surface of the polymer fibers.

**FIGURE 10:**
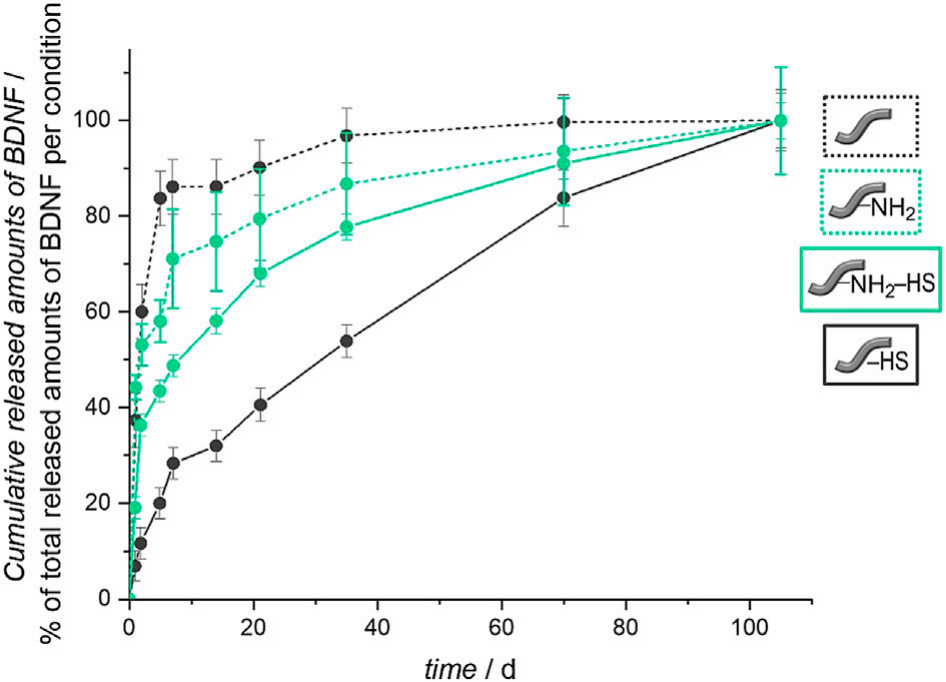
Relative BDNF release curves for unmodified and amino-modified fibers, carrying heparan sulfate or not. The total amount released was set to 100% for each curve.

**FIGURE 11:**
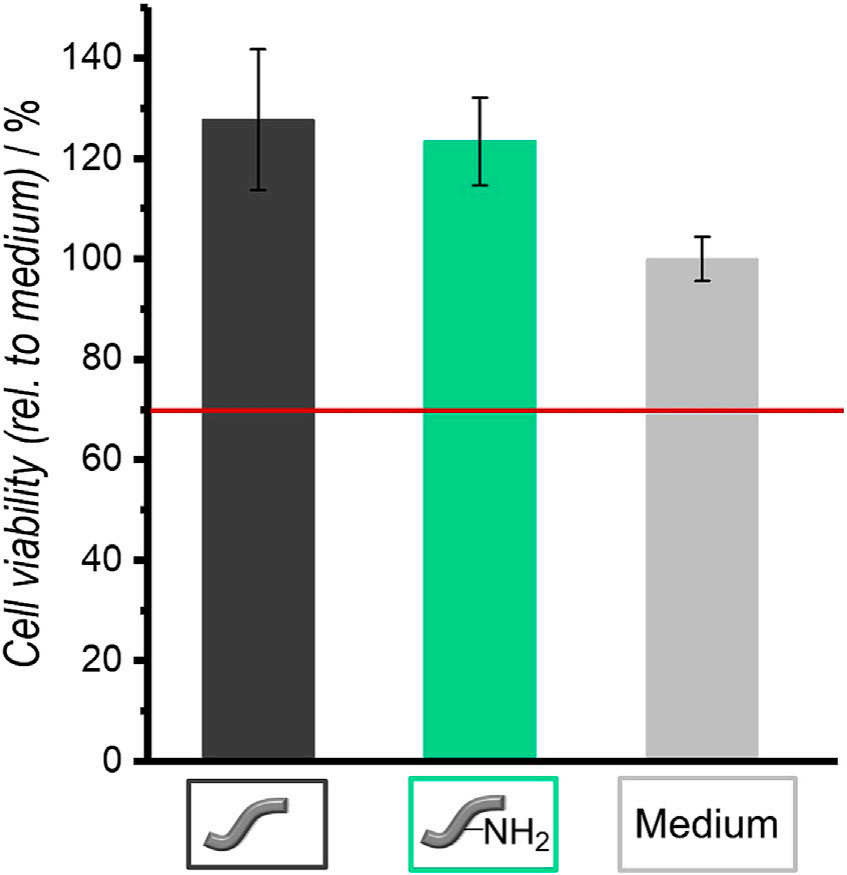
Relative cell viabilities of NIH 3T3 fibroblasts in the presence of unmodified and amino-modified fibers as determined by the NRU assay after an incubation of 4 days. Values are given as mean ± standard error of the mean (*N* = 3).

The general shape of the release curves from HS-coated fibers as depicted in Figure 10 and in Figure 11 can be rationalized with first-order release kinetics where the liberation of the active agent is hindered to some degree, here by the interaction with the host matrix. The differences in the release profiles could be attributed to the differences in interactions between BDNF and the surface of the un- and the amino-modified fibers, both of which are coated with HS, but also the degradation of the polymer fibers themselves could play a role. As the loading efficiency is nearly the same in both cases, it could be assumed that BDNF is attached more strongly to the amino-modified fibers carrying HS, and therefore the release is slower and the total amount released from the surface is less. As the dissolution of the biodegradable polymer fibers under physiological condition (You et al., 2005; Bajgai et al., 2008), like in PBS, can lead to additional release of BDNF, we studied the degradation of these fibers under the conditions employed. As expected, a partial dissolution of the fibers can be observed after release periods of 98 days. SEM investigations show a decrease in the diameters of the fibers and the emerging of cracks over time. These effects are much more pronounced for the amino-modified fibers than for the unmodified fibers, the former ones obviously having been attacked during the modification procedure (compare Figure 5). Furthermore, measurements of the pH value of the surrounding medium show that the decrease in pH value due to the liberation of acidic components during degradation is much less pronounced in the case of the amino-modified fibers (see ESI, Supplementary Figure S1), indicating that basic species (ethylenediamine or derivatives thereof) are set free from the surface of the amino-modified fibers. However, these observations regarding the degradation of the fibers would rather point to a stronger release from the amino-modified fibers, contradicting the finding that lower amounts of released BDNF can be detected from these than from the unmodified fibers. Possibly, BDNF molecules released into the solution due to degradation of the fiber carriers may still carry remnants from the attachment to the fibers, thus rendering such molecules non-detectable by ELISA. In addition, it is possible that the dissolved parts of the polymer fibers (hydroxycarboxylic acids, ethylenediamine, derivatives thereof, and others) could influence the detectability of BDNF in the ELISA (Katsumi Tanaka and Masashi Kumano, 2000). Furthermore, in the interpretation of the results, it has to be taken into account that BDNF molecules may suffer denaturation during the loading and release procedures. However, we consider the latter possibility as less relevant, as the binding to heparan sulfate, the storage on HS as well as the release from HS all resemble natural processes. Moreover, recent studies have shown the successful immobilization on and release of biologically active growth factors from glycosaminoglycans (Sakiyama-Elbert and Hubbell, 2000; Pieper et al., 2002; Singh et al., 2011; Tan et al., 2012). A fibrin-based matrix containing heparin, which is closely related in structure to HS (Rabenstein, 2002), can bind and deliver BDNF (Sakiyama-Elbert and Hubbell, 2000), and poly(*L*-glutamic acid) particles covered with heparin can be used effectively as carrier systems for BDNF delivery (Tan et al., 2012). The vascular endothelial growth factor (VEGF) can be immobilized on and released from a PCL scaffold pre-coated with heparin (Singh et al., 2011). HS was shown to enhance both, the loading and release of the basic fibroblast growth factor (bFGF) on collagen-based matrices (Pieper et al., 2002). Thus, we assume that the interactions between BDNF and the different fiber surfaces have a greater impact on the release profiles than the other factors.

Summarizing, HS-coated fibers offer a sustained long-term delivery of BDNF, independently of whether the fibers were used as received or whether they had been amino-modified. Thus, we chose both types of HS-coated fibers to be employed in cell culture investigations.

### Cell Culture Investigations

#### Cytocompatibility Test With Fibroblasts

Fibroblasts were cultivated in the presence of both types of fibers (one fiber each) to test their cytocompatibility. Figure 11 shows the results of the NRU assay, which was used to determine the cell viability. Unmodified as well as amino-modified fibers are cytocompatible for fibroblasts because their relative cell viability is above 70%. This is a defined criterion for cytocompatibility according to DIN EN ISO 10993-5 (Krämer et al., 2016).

#### Spiral Ganglion Cell Culture Studies With Released BDNF

For their intended application in cochlea implantation, it is important to test whether the polymer fibers provide a suitable carrier system for delivering neurotrophins in a safe and effective way to the inner ear. In the anatomical situation, the target cells, namely spiral ganglion neurons, are not in direct contact with the implant and associated drug delivery systems. Therefore, we carried out spiral ganglion cell cultures not directly in the presence of the fibers, but instead used the supernatants from the release studies of the fibers. We can then assess the neuroprotective effect individually for different time steps of the release. The effect of the released substance is then evaluated by the investigation of the survival rates and neurite lengths of the SGNs.

**FIGURE 12:**
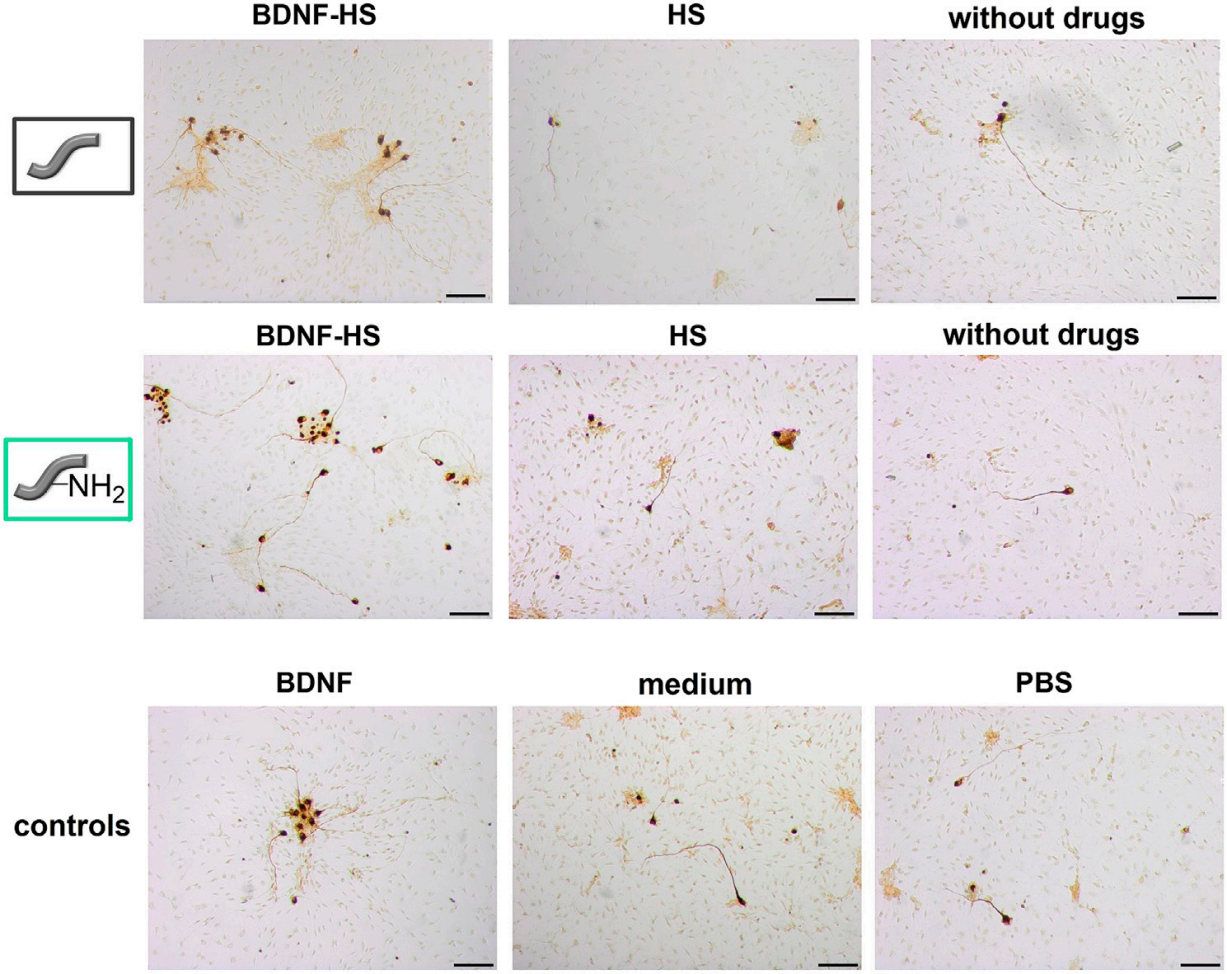
Representative microscopic images of spiral ganglion cell cultures using the supernatants from the two different fiber types, derived from the release experiment of day 1. SGNs are counted as survived neurons when a neurite with a length of at least three soma sizes has grown out of the cell soma. Scale bar 100 µm.

**FIGURE 13:**
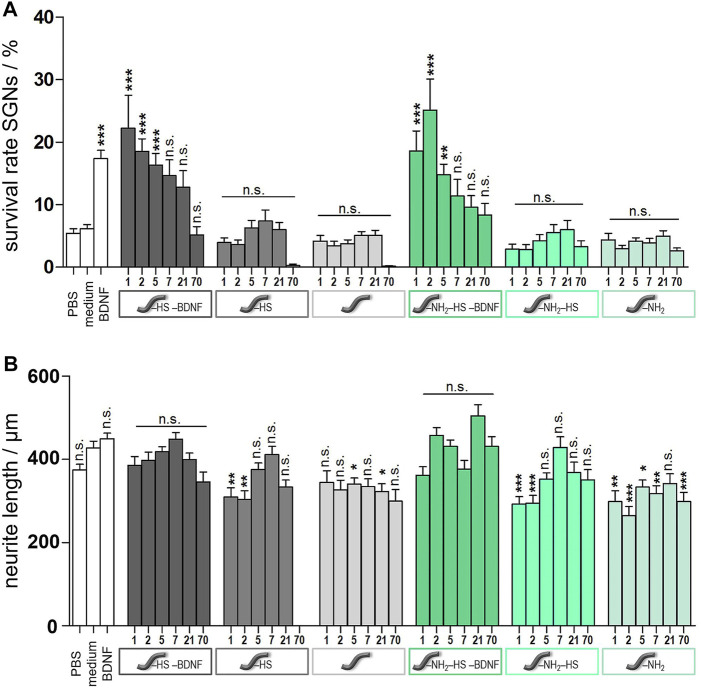
Results from spiral ganglion cell culture studies after cultivation for 2 days. Cells were incubated with the supernatants from the release experiments of either unmodified (grey columns) or amino-modified (green columns) fibers. Fibers carrying BDNF and HS are depicted in the darkest colour shades, those carrying only HS are depicted in medium colour shades, and those not further modified are depicted in the lightest colour shade. Values for controls are shown as white columns (PBS and medium as negative controls, medium with added BDNF as positive control). **(A)** Survival rates of spiral ganglion neurons. **(B)** Neurite lengths of survived SGNs. Values are given as mean ± standard error of the mean (*N* = 3, *n* = 3). Statistical assessment was performed using one-way ANOVA with Tukey’s Multiple comparison test (n.s. = not significant, *p < 0.05; **p < 0.01; ***p < 0.001). Asterisks over the bars indicate the significance of the survival rates of different conditions compared to the medium (serum-free SGN medium) and PBS control (as the results obtained for both cases are very similar to each other) as well as the significance of the neurite lengths of different conditions compared to the medium control.

Spiral ganglion cells were cultured for 2 days in a 1:1 mixture of serum-free SGN medium and the collected supernatants obtained from release experiments with un- and amino-modified fibers, which were either loaded with BDNF or not. After the incubation time, microscopic images were taken (Figure 12). They show differences regarding the neuronal survival and neurite outgrowth. Cells cultured in the presence of the supernatants from the BDNF-loaded fibers (un- and amino-modified) show a high number of surviving neurons with outgrowing neurites. By contrast, less neurons with developed neurites can be found in cell cultures with supernatants derived from the unloaded fibers. Similar results have been obtained when quantifying survival rates of SGNs (Figure 13A; numerical data are listed in Supplementary Table S1 in the ESI). An increased survival rate of SGNs cultivated in supernatants collected from release experiments with BDNF-loaded fibers can be observed in contrast to the control fibers without BDNF loading. This shows that the BDNF released from both types of fibers exerts a distinct neuroprotective effect. The survival rate of SGNs is highly significantly increased for the first three tested time points of the unmodified [1 day 22.3 ± 5.27% (*p* < 0.001); 2 days 18.6 ± 1.99% (*p* < 0.001); 5 days 16.3 ± 1.88% (*p* < 0.001)] and amino-modified [1 day 18.6 ± 3.21% (*p* < 0.001); 2 days 25.2 ± 4.97% (*p* < 0.001); 5 days 14.8 ± 1.71% (*p* < 0.01)] fibers, as compared to the medium (6.13 ± 0.68%) and PBS (5.45 ± 0.70%) controls (the latter being the solution in which the release experiments were carried out). Even in the timeframe between 7 days up to 70 days of release, where the released amounts of BDNF become smaller, the survival rate of SGNs is increased when compared to the negative controls. For the first three time points (day 1–5), the results obtained in case of the BDNF-loaded unmodified as well as of the amino-modified fibers show practically the same survival rate as the positive control (50 ng ml^−1^ BDNF, 17.4% ± 1.32%, n.s.). A reason for this positive result could be the additional slight release of HS from the fibers which we detected (Figure 8). A synenergetic positive effect of the combination of HS and BDNF on neurons can be assumed based on previous investigations (Schulze et al., 2018). According to our data, the presence of HS alone has no neuroprotective effect on the SGNs.

Moreover, one can assume that the actual amount of BDNF present has a proportional effect on the survival rate of the SGNs. As more BDNF is released from the fibers at the first time points (see Figure 9), also the SGN survival rate shows higher values in these cases. This is underlined by the fact that the SGN survival rates are slightly higher in case of the unmodified fibers as compared to the ones of the amino-modified fibers, because somewhat more BDNF is released from the unmodified fibers (see 10). Besides, the stability of the neurotrophin could also play an important role. When using BDNF supernatants collected after a longer period, the BDNF had been incubated for a longer time at 37°C which could affect its conformation and biological activity (Katsumi Tanaka and Masashi Kumano, 2000). Thus, it is possible that supernatants obtained at later time points contain less biologically active BDNF and, thus, the neuroprotective effect is lower.

When comparing all results, the very low SGN survival rates obtained in case of the supernatants that were collected from the unmodified fibers after 70 days stand out. All the survival rates observed for the three different subtypes of unmodified fibers are very low compared to those obtained at earlier time points, respectively. Whereas the unmodified fiber carrying HS and BDNF reaches at least the value of the negative control, virtually no SGNs survive in the presence of supernatants derived from fibers carrying only HS or which are not loaded. We propose that the reason for the low survival rate of the SGNs under these conditions is the low pH value of the supernatants after 70 days (pH = 3.6) due to hydrolytic fiber degradation (see ESI Supplementary Figure S1). During this process, acidic products like (hydroxy-)carboxylic acids are released into the solution and decrease the pH value (You et al., 2005; Bajgai et al., 2008). Phenol red, which is a pH value indicator dye and is contained in the culture medium for the spiral ganglion cells, shows a color change from red to yellow. It is well known that neuronal cells do not survive in acidic medium for a long time and need a well-buffered physiological environment with a neutral pH value (Sung et al., 2004). This effect cannot be observed in case of the amino-modified fibers since the basic amino groups that are released over time as well counteract the acidic groups (pH of solution after release: 4.7, see ESI Supplementary Figure S1).

The measured neurite lengths of the survived SGNs showed comparable values for all samples (Figure 13B), including the controls. Specifically, there is no apparent positive influence of BNDF on the lengths of the neurites. This result is in line with our former observations on a BDNF release system based on nanoporous silica nanoparticles (Schmidt et al., 2018). In general, BDNF is known to mainly have a neuroprotective influence, countering neuron degradation (Budenz et al., 2015; Xiao, 2016; Schmidt et al., 2018).

Our results are promising for the application of BDNF-loaded polymer fibers as a release system on a cochlea implant. Overall, the released BDNF from both types of fibers has a clear neuroprotective effect on SGNs. We could show that the amount of BDNF has an impact on the SGN survival rate, but also smaller amounts of released BDNF show a remarkable effect on the survival rate of the SGNs. The delivery prevails over long timescales and has not finished after the observed timeframe of 110 days.

Our results are corroborated by previous studies that showed that biologically active BDNF can be released from glycosaminoglycan structures (Sakiyama-Elbert and Hubbell, 2000; Tan et al., 2012), and that a neuroprotective effect of released BDNF towards SGNs can be observed even with only small quantities (ng/pg range) of the growth factor *in vitro* and *in vivo* (Warnecke et al., 2012; Schmidt et al., 2018; Scheper et al., 2019). Also, BDNF delivered by heparin-coated poly (L-glutamic acid) particles in the inner ear of a guinea pig led to an enhanced neuronal survival (Tan et al., 2012).

## Conclusion

In this study, we showed that biodegradable polymer fibers pre-coated with heparan sulfate are an effective matrix for the delivery of neurotrophins like BDNF to enhance neuronal survival *in vitro*. Both, unmodified and amino-modified fibers showed a good general cytocompatibility towards NIH 3T3 fibroblasts and could be loaded with HS. The loading and release of biologically relevant amounts of BDNF to the HS-coated fibers was demonstrated. More BDNF was released from the unmodified fibers, but both systems showed a sustained release over a period of at least 110 days. Thus, they are a promising material for a long-term delivery of neurotrophins to prevent neuronal degeneration. *In vitro* experiments with SGNs revealed a significant neuroprotective effect of the released BDNF from both types of fibers. In the future, release experiments could involve the presence of an ECM-degrading enzyme (e.g., heparanase for the digestion of heparan sulfate) in order to more closely mimic *in vivo* release conditions. Degradation of the artificial ECM mimic we employ might change the release kinetics.

The fibers constructed here have clearly shown their applicability as potential CI-associated carrier systems for HS to deliver growth factors like BDNF. Even though not investigated in this study, a BDNF concentration gradient emanating from the polymer fibers could be a conceivable method for not only enhancing the survival rate of the SGNs, but also for guiding their neurites along the fibers and finally towards the surface of the implant. Several studies have demonstrated neuronal growth towards a neurotrophin concentration gradient (Xie et al., 2012; Li H. et al., 2017; Yilmaz-Bayraktar et al., 2020). Xie et al. (2012) showed that a BDNF reservoir leads to a long distance axonal growth of hippocampal neurons along an ordered channel system. Further studies support the successful guidance of SGNs along ordered structures and their positive effect on the length of the neurites (Leigh et al., 2017; Yilmaz-Bayraktar et al., 2020). Whereas other studies have reported a neuroregenerative effect of BDNF, based on increasing lengths of neurites (Gao et al., 2016; McGregor and English, 2019), we were not able to confirm this in the present work nor in former BDNF delivery experiments (Schmidt et al., 2018). In this context, the use of other growth factors with stronger effects on neurite outgrowth can be envisaged, still based on the general make-up of the fibers presented here.

With regard to the further development of the polymer fibers to establish a neuronal guidance scaffold in the inner ear, the installation of other features may become important. For example, once the neurites have reached the fibers, it will be important to configure a sensitive equilibrium between attachment of the neurites onto the fibers—which can be facilitated by immobilizing neural adhesion proteins on the fiber surface—and permissive structures which encourage further growth along the fiber to finally reach the implant body. For this purpose, the amino modification of the fiber surface may be more important than in the present case (where the amino-modified fibers deliver less but sufficient amounts of BDNF), as this modification offers chemically more reactive sites than the unmodified fibers.

## Data Availability

The original contributions presented in the study are included in the article/Supplementary Material, further inquiries can be directed to the corresponding authors.

## References

[B1] Apfel S. C. Wright D. E. Wiideman A. M. Dormia C. Snider W. D. Kessler J. A. (1996). Nerve Growth Factor Regulates the Expression of Brain-Derived Neurotrophic Factor mRNA in the Peripheral Nervous System. Mol. Cell Neurosci. 7, 134–142. 10.1006/mcne.1996.0010 8731481

[B2] Bajgai M. P. Kim K.-W. Chandra Parajuli D. Yoo Y. C. Kim W. D. Khil M.-S. (2008). *In Vitro* hydrolytic Degradation of Poly(ɛ-Caprolactone) Grafted Dextran Fibers and Films. Polym. Degrad. Stab. 93, 2172–2179. 10.1016/j.polymdegradstab.2008.08.002

[B3] Barde Y. A. Edgar D. Thoenen H. (1982). Purification of a New Neurotrophic Factor from Mammalian Brain. EMBO J. 1, 549–553. 10.1002/j.1460-2075.1982.tb01207.x 7188352 PMC553086

[B4] Bezwada R. S. Jamiolkowski D. D. Lee I.-Y. Agarwal V. Persivale J. Trenka-Benthin S. (1995). Monocryl Suture, a New Ultra-pliable Absorbable Monofilament Suture. Biomaterials 16, 1141–1148. 10.1016/0142-9612(95)93577-Z 8562789

[B5] Budenz C. L. Wong H. T. Swiderski D. L. Shibata S. B. Pfingst B. E. Raphael Y. (2015). Differential Effects of AAV.BDNF and AAV.Ntf3 in the Deafened Adult Guinea Pig Ear. Sci. Rep. 5, 8619. 10.1038/srep08619 25726967 PMC4649680

[B6] Budweiser J. (2015). Hydrolyseverhalten der synthetischen Phospholipide DPPGx (x=1,2,3) im Vergleich zu DPPC in thermosensitiven Liposomen.

[B7] Cacicol Augentropfen (2019). Available at: https://www.sparmedo.de/produktinfo/cacicol-augentropfen-10039463/ (Accessed September 9, 2020).

[B8] Cai Y. Edin F. Jin Z. Alexsson A. Gudjonsson O. Liu W. (2016). Strategy towards Independent Electrical Stimulation from Cochlear Implants: Guided Auditory Neuron Growth on Topographically Modified Nanocrystalline diamond. Acta Biomater. 31, 211–220. 10.1016/j.actbio.2015.11.021 26593784

[B9] Carraway K. L. Koshland D. E. (1972). [56] Carbodiimide Modification of Proteins. Methods Enzymol. 25, 616–623. 10.1016/S0076-6879(72)25060-1 23014445

[B10] Choi J. S. Leong K. W. Yoo H. S. (2008). *In Vivo* wound Healing of Diabetic Ulcers Using Electrospun Nanofibers Immobilized with Human Epidermal Growth Factor (EGF). Biomaterials 29, 587–596. 10.1016/j.biomaterials.2007.10.012 17997153

[B11] Chuang C. Y. Lord M. S. Melrose J. Rees M. D. Knox S. M. Freeman C. (2010). Heparan Sulfate-dependent Signaling of Fibroblast Growth Factor 18 by Chondrocyte-Derived Perlecan. Biochemistry 49, 5524–5532. 10.1021/bi1005199 20507176 PMC2900151

[B12] Dhanasingh A. Jolly C. (2017). An Overview of Cochlear Implant Electrode Array Designs. Hearing Res. 356, 93–103. 10.1016/j.heares.2017.10.005 29102129

[B13] Dinbergs I. D. Brown L. Edelman E. R. (1996). Cellular Response to Transforming Growth Factor-Β1 and Basic Fibroblast Growth Factor Depends on Release Kinetics and Extracellular Matrix Interactions. J. Biol. Chem. 271, 29822–29829. 10.1074/jbc.271.47.29822 8939921

[B14] Duan M. L. Ulfendahl M. Laurell G. Counter A. S. Pyykkö I. Borg E. (2002). Protection and Treatment of Sensorineural Hearing Disorders Caused by Exogenous Factors: Experimental Findings and Potential Clinical Application. Hearing Res. 169, 169–178. 10.1016/S0378-5955(02)00484-7 12121749

[B15] El Kechai N. Agnely F. Mamelle E. Nguyen Y. Ferrary E. Bochot A. (2015). Recent Advances in Local Drug Delivery to the Inner Ear. Int. J. Pharmaceutics 494, 83–101. 10.1016/j.ijpharm.2015.08.015 26260230

[B16] Endo T. Nakagawa T. Kita T. Iguchi F. Kim T.-S. Tamura T. (2005). Novel Strategy for Treatment of Inner Ears Using a Biodegradable Gel. The Laryngoscope 115, 2016–2020. 10.1097/01.mlg.0000183020.32435.59 16319616

[B17] Frazza E. J. Schmitt E. E. (1971). A New Absorbable Suture. J. Biomed. Mater. Res. 5, 43–58. 10.1002/jbm.820050207 5575328

[B18] Freed L. E. Vunjak-Novakovic G. Biron R. J. Eagles D. B. Lesnoy D. C. Barlow S. K. (1994). Biodegradable Polymer Scaffolds for Tissue Engineering. Nat. Biotechnol. 12, 689–693. 10.1038/nbt0794-689 7764913

[B19] Gao M. Lu P. Lynam D. Bednark B. Campana W. M. Sakamoto J. (2016). BDNF Gene Delivery within and beyond Templated Agarose Multi-Channel Guidance Scaffolds Enhances Peripheral Nerve Regeneration. J. Neural Eng. 13, 066011–066019. 10.1088/1741-2560/13/6/066011 27762235 PMC12529108

[B20] Géral C. Angelova A. Lesieur S. (2013). From Molecular to Nanotechnology Strategies for Delivery of Neurotrophins: Emphasis on Brain-Derived Neurotrophic Factor (BDNF). Pharmaceutics 5, 127–167. 10.3390/pharmaceutics5010127 24300402 PMC3834942

[B21] Gillespie L. N. Clark G. M. Bartlett P. F. Marzella P. L. (2003). BDNF-induced Survival of Auditory Neurons *In Vivo*: Cessation of Treatment Leads to Accelerated Loss of Survival Effects. J. Neurosci. Res. 71, 785–790. 10.1002/jnr.10542 12605404

[B22] Gillespie L. N. Clark G. M. Bartlett P. F. Marzella P. L. (2001). LIF Is More Potent Than BDNF in Promoting Neurite Outgrowth of Mammalian Auditory Neurons *In Vitro* . Neuroreport 12, 275–279. 10.1097/00001756-200102120-00019 11209934

[B23] Gillespie L. N. Shepherd R. K. (2005). Clinical Application of Neurotrophic Factors: the Potential for Primary Auditory Neuron protection. Eur. J. Neurosci. 22, 2123–2133. 10.1111/j.1460-9568.2005.04430.x 16262651 PMC1831824

[B24] Gloria A. Causa F. Russo T. Battista E. Della Moglie R. Zeppetelli S. (2012). Three-Dimensional Poly(ε-Caprolactone) Bioactive Scaffolds with Controlled Structural and Surface Properties. Biomacromolecules 13, 3510–3521. 10.1021/bm300818y 23030686

[B25] Gospodarowicz D. Cheng J. (1986). Heparin Protects Basic and Acidic FGF from Inactivation. J. Cel. Physiol. 128, 475–484. 10.1002/jcp.1041280317 3528177

[B26] Günzler H. Gremlich H.-U. (2003). IR-spektroskopie. Wiley-VCH GmbH & Co. KGaA.

[B27] Hackelberg S. Tuck S. J. He L. Rastogi A. White C. Liu L. (2017). Nanofibrous Scaffolds for the Guidance of Stem Cell-Derived Neurons for Auditory Nerve Regeneration. PLoS One 12, e0180427. 10.1371/journal.pone.0180427 28672008 PMC5495534

[B28] Häcker U. Nybakken K. Perrimon N. (2005). Heparan Sulphate Proteoglycans: the Sweet Side of Development. Nat. Rev. Mol. Cel Biol. 6, 530–541. 10.1038/nrm1681 16072037

[B29] Hearing loss is a big worldwide problem (2016). Available at: https://www.hear-it.org/hearing-loss-big-worldwide-problem (Accessed August 3, 2020).

[B30] Hesse M. Meier H. Zeeh B. (2005). Spektroskopische Methoden in der organischen Chemie.

[B31] Hoffman A. S. Schmer G. Harris C. Kraft W. G. (1972). Covalent Binding of Biomolecules to Radiation-Grafted Hydrogels on Inert Polymer Surfaces. ASAIO J. 18, 10–16. 10.1097/00002480-197201000-00003 4679861

[B32] Horne M. K. Nisbet D. R. Forsythe J. S. Parish C. L. (2010). Three-Dimensional Nanofibrous Scaffolds Incorporating Immobilized BDNF Promote Proliferation and Differentiation of Cortical Neural Stem Cells. Stem Cell Develop. 19, 843–852. 10.1089/scd.2009.0158 19831634

[B33] Houshyar S. Bhattacharyya A. Shanks R. (2019). Peripheral Nerve Conduit: Materials and Structures. ACS Chem. Neurosci. 10, 3349–3365. 10.1021/acschemneuro.9b00203 31273975

[B34] Ito Y. Sisido M. Imanishi Y. (1986). Synthesis and Antithrombogenicity of Anionic Polyurethanes and Heparin-Bound Polyurethanes. J. Biomed. Mater. Res. 20, 1157–1177. 10.1002/jbm.820200808 3782177

[B35] Kalaska B. Miklosz J. Kamiński K. Musielak B. Yusa S.-I. Pawlak D. (2019). The Neutralization of Heparan Sulfate by Heparin-Binding Copolymer as a Potential Therapeutic Target. RSC Adv. 9, 3020–3029. 10.1039/C8RA09724K PMC9059929 35518950

[B36] Kang I.-K. Kwon O. H. Lee Y. M. Sung Y. K. (1996). Preparation and Surface Characterization of Functional Group-Grafted and Heparin-Immobilized Polyurethanes by Plasma Glow Discharge. Biomaterials 17, 841–847. 10.1016/0142-9612(96)81422-0 8730969

[B37] Katsumi Tanaka T. Masashi Kumano M. (2000). Stable Pharmaceutical Composition of BDNF.

[B38] Konerding W. S. Janssen H. Hubka P. Tornøe J. Mistrik P. Wahlberg L. (2017). Encapsulated Cell Device Approach for Combined Electrical Stimulation and Neurotrophic Treatment of the Deaf Cochlea. Hearing Res. 350, 110–121. 10.1016/j.heares.2017.04.013 28463804

[B39] Krämer M. Schilling M. Eifler R. Hering B. Reifenrath J. Besdo S. (2016). Corrosion Behavior, Biocompatibility and Biomechanical Stability of a Prototype Magnesium-Based Biodegradable Intramedullary Nailing System. Mater. Sci. Eng. C 59, 129–135. 10.1016/j.msec.2015.10.006 26652357

[B40] Kranz K. Warnecke A. Lenarz T. Durisin M. Scheper V. (2014). Phosphodiesterase Type 4 Inhibitor Rolipram Improves Survival of Spiral Ganglion Neurons *In Vitro* . PLoS One 9, e92157. 10.1371/journal.pone.0092157 24642701 PMC3958480

[B41] Laske C. Eschweiler G. W. (2006). Brain-derived Neurotrophic Factor. Nervenarzt 77, 523–537. 10.1007/s00115-005-1971-0 16078056

[B42] Leigh B. L. Truong K. Bartholomew R. Ramirez M. Hansen M. R. Guymon C. A. (2017). Tuning Surface and Topographical Features to Investigate Competitive Guidance of Spiral Ganglion Neurons. ACS Appl. Mater. Inter. 9, 31488–31496. 10.1021/acsami.7b09258 PMC6341486 28841276

[B43] Lenarz T. (1998). Cochlea-Implantat: ein praktischer Leitfaden für die Versorgung von Kindern und Erwachsenen. Berlin Heidelberg: Springer Berlin Heidelberg.

[B44] Li H. Edin F. Hayashi H. Gudjonsson O. Danckwardt-Lillieström N. Engqvist H. (2017a). Guided Growth of Auditory Neurons: Bioactive Particles towards Gapless Neural - Electrode Interface. Biomaterials 122, 1–9. 10.1016/j.biomaterials.2016.12.020 28107660

[B45] Li L. Chao T. Brant J. O'Malley B. Tsourkas A. Li D. (2017b). Advances in Nano-Based Inner Ear Delivery Systems for the Treatment of Sensorineural Hearing Loss. Adv. Drug Deliv. Rev. 108, 2–12. 10.1016/j.addr.2016.01.004 26796230 PMC4940320

[B46] Li X. Su Y. Liu S. Tan L. Mo X. Ramakrishna S. (2010). Encapsulation of Proteins in Poly(l-Lactide-Co-Caprolactone) Fibers by Emulsion Electrospinning. Colloids Surf. B: Biointerfaces 75, 418–424. 10.1016/j.colsurfb.2009.09.014 19836931

[B47] Lin Y.-S. Tsai C.-P. Huang H.-Y. Kuo C.-T. Hung Y. Huang D.-M. (2005). Well-Ordered Mesoporous Silica Nanoparticles as Cell Markers. Chem. Mater. 17, 4570–4573. 10.1021/cm051014c

[B48] Macintosh F. C. (1941). A Colorimetric Method for the Standardization of Heparin Preparations. Biochem. J. 35, 776–782. 10.1042/bj0350776 16747363 PMC1265562

[B49] McDonald N. Q. Chao M. V. (1995). Structural Determinants of Neurotrophin Action. J. Biol. Chem. 270, 19669–19672. 10.1074/jbc.270.34.19669 7649974

[B50] McGregor C. E. English A. W. (2019). The Role of BDNF in Peripheral Nerve Regeneration: Activity-dependent Treatments and Val66Met. Front. Cel. Neurosci. 12. Article 522. 10.3389/fncel.2018.00522 PMC6336700 30687012

[B51] Meltser I. Tahera Y. Canlon B. (2010). Differential Activation of Mitogen-Activated Protein Kinases and Brain-Derived Neurotrophic Factor after Temporary or Permanent Damage to a Sensory System. Neuroscience 165, 1439–1446. 10.1016/j.neuroscience.2009.11.025 19925854

[B52] Meneghetti M. C. Z. Hughes A. J. Rudd T. R. Nader H. B. Powell A. K. Yates E. A. (2015). Heparan Sulfate and Heparin Interactions with Proteins. J. R. Soc. Interf. 12, 20150589. 10.1098/rsif.2015.0589 PMC4614469 26289657

[B53] Middleton J. C. Tipton A. J. (2000). Synthetic Biodegradable Polymers as Orthopedic Devices. Biomaterials 21, 2335–2346. 10.1016/S0142-9612(00)00101-0 11055281

[B54] Miller J. M. Chi D. H. O'Keeffe L. J. Kruszka P. Raphael Y. Altschuler R. A. (1997). Neurotrophins Can Enhance Spiral Ganglion Cell Survival after Inner Hair Cell Loss. Int. J. Develop. Neurosci. 15, 631–643. 10.1016/S0736-5748(96)00117-7 9263039

[B55] Mohtaram N. K. Montgomery A. Willerth S. M. (2013). Biomaterial-based Drug Delivery Systems for the Controlled Release of Neurotrophic Factors. Biomed. Mater. 8, 022001. 10.1088/1748-6041/8/2/022001 23385544

[B56] Nacher-Soler G. Garrido J. M. Rodríguez-Serrano F. (2019). Hearing Regeneration and Regenerative Medicine: Present and Future Approaches. aoms 15, 957–967. 10.5114/aoms.2019.86062 PMC6657260 31360190

[B57] O'Leary P. D. Hughes R. A. (2003). Design of Potent Peptide Mimetics of Brain-Derived Neurotrophic Factor. J. Biol. Chem. 278, 25738–25744. 10.1074/jbc.M303209200 12730196

[B58] Okolicsanyi R. K. Griffiths L. R. Haupt L. M. (2014). Mesenchymal Stem Cells, Neural Lineage Potential, Heparan Sulfate Proteoglycans and the Matrix. Develop. Biol. 388, 1–10. 10.1016/j.ydbio.2014.01.024 24509075

[B59] Pfingst B. E. Zhou N. Colesa D. J. Watts M. M. Strahl S. B. Garadat S. N. (2015). Importance of Cochlear Health for Implant Function. Hearing Res. 322, 77–88. 10.1016/j.heares.2014.09.009 PMC4377117 25261772

[B60] Pieper J. S. Hafmans T. van Wachem P. B. van Luyn M. J. A. Brouwer L. A. Veerkamp J. H. (2002). Loading of Collagen-Heparan Sulfate Matrices with bFGF Promotes Angiogenesis and Tissue Generation in Rats. J. Biomed. Mater. Res. 62, 185–194. 10.1002/jbm.10267 12209938

[B61] Pieper J. S. Hafmans T. Veerkamp J. H. van Kuppevelt T. H. (2000a). Development of Tailor-Made Collagen-Glycosaminoglycan Matrices: EDC/NHS Crosslinking, and Ultrastructural Aspects. Biomaterials 21, 581–593. 10.1016/S0142-9612(99)00222-7 10701459

[B62] Pieper J. S. van Wachem P. B. van Luyn M. J. A. Brouwer L. A. Hafmans T. Veerkamp J. H. (2000b). Attachment of Glycosaminoglycans to Collagenous Matrices Modulates the Tissue Response in Rats. Biomaterials 21, 1689–1699. 10.1016/S0142-9612(00)00052-1 10905410

[B63] Rabenstein D. L. (2002). Heparin and Heparan Sulfate: Structure and Function. Nat. Prod. Rep. 19, 312–331. 10.1039/b100916h 12137280

[B64] Rask-Andersen H. Liu W. (2015). Auditory Nerve Preservation and Regeneration in Man: Relevance for Cochlear Implantation. Neural Regen. Res. 10, 710. 10.4103/1673-5374.156963 26109941 PMC4468758

[B65] Rejali D. Lee V. A. Abrashkin K. A. Humayun N. Swiderski D. L. Raphael Y. (2007). Cochlear Implants and *Ex Vivo* BDNF Gene Therapy Protect Spiral Ganglion Neurons. Hearing Res. 228, 180–187. 10.1016/j.heares.2007.02.010 PMC2692458 17416474

[B66] Roghani M. Moscatelli D. (1992). Basic Fibroblast Growth Factor Is Internalized through Both Receptor-Mediated and Heparan Sulfate-Mediated Mechanisms. J. Biol. Chem. 267, 22156–22162. 10.1016/s0021-9258(18)41648-1 1429568

[B67] Rohman G. Baker S. C. Southgate J. Cameron N. R. (2009). Heparin Functionalisation of Porous PLGA Scaffolds for Controlled, Biologically Relevant Delivery of Growth Factors for Soft Tissue Engineering. J. Mater. Chem. 19, 9265. 10.1039/b911625g

[B68] Ruoslahti E. Yamaguchi Y. (1991). Proteoglycans as Modulators of Growth Factor Activities. Cell 64, 867–869. 10.1016/0092-8674(91)90308-l 2001586

[B69] Rüttiger L. Panford-Walsh R. Schimmang T. Tan J. Zimmermann U. Rohbock K. (2007). BDNF mRNA Expression and Protein Localization Are Changed in Age-Related Hearing Loss. Neurobiol. Aging 28, 586–601. 10.1016/j.neurobiolaging.2006.02.008 16580094

[B70] Ryan A. F. Wittig J. Evans A. Dazert S. Mullen L. (2006). Environmental Micropatterning for the Study of Spiral Ganglion Neurite Guidance. Audiol. Neurotol. 11, 134–143. 10.1159/000090686 16439836

[B71] Sakiyama-Elbert S. E. Hubbell J. A. (2000). Controlled Release of Nerve Growth Factor from a Heparin-Containing Fibrin-Based Cell Ingrowth Matrix. J. Controlled Release 69, 149–158. 10.1016/S0168-3659(00)00296-0 11018553

[B72] Scheper V. Hoffmann A. Gepp M. M. Schulz A. Hamm A. Pannier C. (2019). Stem Cell Based Drug Delivery for Protection of Auditory Neurons in a Guinea Pig Model of Cochlear Implantation. Front. Cel. Neurosci. 13, 1–16. 10.3389/fncel.2019.00177 PMC6527816 31139049

[B73] Schilder A. G. M. Su M. P. Blackshaw H. Lustig L. Staecker H. Lenarz T. (2019). Hearing Protection, Restoration, and Regeneration: An Overview of Emerging Therapeutics for Inner Ear and Central Hearing Disorders. Otol. Neurotol. 40, 559–570. 10.1097/MAO.0000000000002194 31083073

[B74] Schlessinger J. Lax I. Lemmon M. (1995). Regulation of Growth Factor Activation by Proteoglycans: What Is the Role of the Low Affinity Receptors? Cell 83, 357–360. 10.1016/0092-8674(95)90112-4 8521464

[B75] Schlie-Wolter S. Deiwick A. Fadeeva E. Paasche G. Lenarz T. Chichkov B. N. (2013). Topography and Coating of Platinum Improve the Electrochemical Properties and Neuronal Guidance. ACS Appl. Mater. Inter. 5, 1070–1077. 10.1021/am3028487 23327880

[B76] Schmidt C. Thull R. (2003). Charakterisierung Heparin- Beschichteter Titandioxide. Biomaterialien 4, 11–16. 10.1515/biomat.2003.4.1.38

[B77] Schmidt N. Schulze J. Warwas D. P. Ehlert N. Lenarz T. Warnecke A. (2018). Long-term Delivery of Brain-Derived Neurotrophic Factor (BDNF) from Nanoporous Silica Nanoparticles Improves the Survival of Spiral Ganglion Neurons *In Vitro* . PLoS One 13, e0194778. 10.1371/journal.pone.0194778 29584754 PMC5870973

[B78] Schulze J. Sasse S. Prenzler N. Staecker H. Mellott A. J. Roemer A. (2018). Microenvironmental Support for Cell Delivery to the Inner Ear. Hearing Res. 368, 109–122. 10.1016/j.heares.2018.06.015 29945803

[B79] Senn P. Roccio M. Hahnewald S. Frick C. Kwiatkowska M. Ishikawa M. (2017). NANOCI-nanotechnology Based Cochlear Implant with Gapless Interface to Auditory Neurons. Otol. Neurotol. 38, e224–e231. 10.1097/MAO.0000000000001439 28806330 PMC5559190

[B80] Shepherd R. K. Coco A. Epp S. B. (2008). Neurotrophins and Electrical Stimulation for protection and Repair of Spiral Ganglion Neurons Following Sensorineural Hearing Loss. Hearing Res. 242, 100–109. 10.1016/j.heares.2007.12.005 PMC2630855 18243608

[B81] Shibata S. B. Budenz C. L. Bowling S. A. Pfingst B. E. Raphael Y. (2011). Nerve Maintenance and Regeneration in the Damaged Cochlea. Hearing Res. 281, 56–64. 10.1016/j.heares.2011.04.019 PMC3196294 21596129

[B82] Singer W. Panford-Walsh R. Knipper M. (2014). The Function of BDNF in the Adult Auditory System. Neuropharmacology 76, 719–728. 10.1016/j.neuropharm.2013.05.008 23688926

[B83] Singh S. Wu B. M. Dunn J. C. Y. (2011). The Enhancement of VEGF-Mediated Angiogenesis by Polycaprolactone Scaffolds with Surface Cross-Linked Heparin. Biomaterials 32, 2059–2069. 10.1016/j.biomaterials.2010.11.038 21147501 PMC3030207

[B84] Sommer A. Rifkin D. B. (1989). Interaction of Heparin with Human Basic Fibroblast Growth Factor: Protection of the Angiogenic Protein from Proteolytic Degradation by a Glycosaminoglycan. J. Cel. Physiol. 138, 215–220. 10.1002/jcp.1041380129 2910884

[B85] Spearman S. S. Rivero I. V. Abidi N. (2014). Influence of Polycaprolactone/polyglycolide Blended Electrospun Fibers on the Morphology and Mechanical Properties of Polycaprolactone. J. Appl. Polym. Sci. 131, a–n. 10.1002/app.40224

[B86] Staecker H. Gabaizadeh R. Federoff H. Water T. R. V. D. (1998). Brain-derived Neurotrophic Factor Gene Therapy Prevents Spiral Ganglion Degeneration after Hair Cell Loss. Otolaryngol. Head Neck Surg. 119, 7–13. 10.1016/s0194-5998(98)70194-9 9674508

[B87] Stieghorst J. Tran B. N. Hadeler S. Beckmann D. Doll T. (2016). Hydrogel-Based Actuation for Modiolar Hugging Cochlear Implant Electrode Arrays. Proced. Eng. 168, 1529–1532. 10.1016/j.proeng.2016.11.453 26863644

[B88] Stöver T. Lenarz T. (2009). Biomaterials in Cochlear Implants. Laryngorhinootologie 88, S12–S31. 10.3205/cto00006210.1055/s-0028-1119552 19353453

[B89] Sung H.-J. Meredith C. Johnson C. Galis Z. S. (2004). The Effect of Scaffold Degradation Rate on Three-Dimensional Cell Growth and Angiogenesis. Biomaterials 25, 5735–5742. 10.1016/j.biomaterials.2004.01.066 15147819

[B90] Swan E. E. L. Mescher M. J. Sewell W. F. Tao S. L. Borenstein J. T. (2008). Inner Ear Drug Delivery for Auditory Applications. Adv. Drug Deliv. Rev. 60, 1583–1599. 10.1016/j.addr.2008.08.001 18848590 PMC2657604

[B91] Szentivanyi A. Chakradeo T. Zernetsch H. Glasmacher B. (2011). Electrospun Cellular Microenvironments: Understanding Controlled Release and Scaffold Structure. Adv. Drug Deliv. Rev. 63, 209–220. 10.1016/j.addr.2010.12.002 21145932

[B92] Tan J. Wang Y. Yip X. Glynn F. Shepherd R. K. Caruso F. (2012). Nanoporous Peptide Particles for Encapsulating and Releasing Neurotrophic Factors in an Animal Model of Neurodegeneration. Adv. Mater. 24, 3362–3366. 10.1002/adma.201200634 22610659 PMC3543853

[B93] Wang Y. Wise A. K. Tan J. Maina J. W. Shepherd R. K. Caruso F. (2014). Mesoporous Silica Supraparticles for Sustained Inner-Ear Drug Delivery. Small 10, 4244–4248. 10.1002/smll.201401767 25099026

[B94] Warnecke A. Sasse S. Wenzel G. I. Hoffmann A. Gross G. Paasche G. (2012). Stable Release of BDNF from the Fibroblast Cell Line NIH3T3 Grown on Silicone Elastomers Enhances Survival of Spiral Ganglion Cells *In Vitro* and *In Vivo* . Hearing Res. 289, 86–97. 10.1016/j.heares.2012.04.007 22564255

[B95] Wefstaedt P. Scheper V. Lenarz T. Stöver T. (2005). Brain-derived Neurotrophic Factor/glial Cell Line-Derived Neurotrophic Factor Survival Effects on Auditory Neurons Are Not Limited by Dexamethasone. Neuroreport 16, 2011–2014. 10.1097/00001756-200512190-00008 16317344

[B96] Who (2021). Deafness and Hearing Loss. Available at: https://www.who.int/news-room/fact-sheets/detail/deafness-and-hearing-loss (Accessed June 8, 2021).

[B97] Williams S. Neumann A. Bremer I. Su Y. Dräger G. Kasper C. (2015). Nanoporous Silica Nanoparticles as Biomaterials: Evaluation of Different Strategies for the Functionalization with Polysialic Acid by Step-by-step Cytocompatibility Testing. J. Mater. Sci. Mater. Med. 26, 125. 10.1007/s10856-015-5409-3 25690616

[B98] Wilson B. S. Dorman M. F. (2018). “Stimulation for the Return of Hearing,” in “Stimulation for the Return of Hearing,” in *Neuromodulation* . Editors Krames E. S. Hunter Peckham P. Rezai A. R. (Elsevier), 1209–1221. 10.1016/B978-0-12-805353-9.00100-5

[B99] Wilson B. S. (2017). The Cochlear Implant and Possibilities for Narrowing the Remaining Gaps between Prosthetic and normal Hearing. World J. Otorhinolaryngol. - Head Neck Surg. 3, 200–210. 10.1016/j.wjorl.2017.12.005 29780963 PMC5956133

[B100] Wise A. K. Richardson R. Hardman J. Clark G. O'Leary S. (2005). Resprouting and Survival of guinea Pig Cochlear Neurons in Response to the Administration of the Neurotrophins Brain-Derived Neurotrophic Factor and Neurotrophin-3. J. Comp. Neurol. 487, 147–165. 10.1002/cne.20563 15880560

[B101] Wise A. K. Tan J. Wang Y. Caruso F. Shepherd R. K. (2016). Improved Auditory Nerve Survival with Nanoengineered Supraparticles for Neurotrophin Delivery into the Deafened Cochlea. PLoS One 11, e0164867. 10.1371/journal.pone.0164867 27788219 PMC5082918

[B102] Woodruff M. A. Hutmacher D. W. (2010). The Return of a Forgotten Polymer-Polycaprolactone in the 21st century. Prog. Polym. Sci. 35, 1217–1256. 10.1016/j.progpolymsci.2010.04.002

[B103] Xiao N. (2016). Neurotrophic Factors: Promising Candidates in Tissue Regeneration. Neural Regen. Res. 11, 735. 10.4103/1673-5374.182696 27335553 PMC4904460

[B104] Xie W. Zhang K. Cui B. (2012). Functional Characterization and Axonal Transport of Quantum Dot Labeled BDNF. Integr. Biol. 4, 953. 10.1039/c2ib20062g PMC3462492 22772872

[B105] Yilmaz-Bayraktar S. Schwieger J. Scheper V. Lenarz T. Böer U. Kreienmeyer M. (2020). Decellularized Equine Carotid Artery Layers as Matrix for Regenerated Neurites of Spiral Ganglion Neurons. Int. J. Artif. Organs 43, 332–342. 10.1177/0391398819868481 31434531 PMC7221869

[B106] Yoo H. S. Kim T. G. Park T. G. (2009). Surface-functionalized Electrospun Nanofibers for Tissue Engineering and Drug Delivery. Adv. Drug Deliv. Rev. 61, 1033–1042. 10.1016/j.addr.2009.07.007 19643152

[B107] You Y. Min B.-M. Lee S. J. Lee T. S. Park W. H. (2005). *In Vitro* degradation Behavior of Electrospun Polyglycolide, Polylactide, and Poly(lactide-Co-Glycolide). J. Appl. Polym. Sci. 95, 193–200. 10.1002/app.21116

[B108] Young A. T. Cornwell N. Daniele M. A. (2018). Neuro‐Nano Interfaces: Utilizing Nano‐Coatings and Nanoparticles to Enable Next‐Generation Electrophysiological Recording, Neural Stimulation, and Biochemical Modulation. Adv. Funct. Mater. 28, 1700239. 10.1002/adfm.201700239 33867903 PMC8049593

[B109] Zhu Y. Gao C. Liu X. Shen J. (2002). Surface Modification of Polycaprolactone Membrane via Aminolysis and Biomacromolecule Immobilization for Promoting Cytocompatibility of Human Endothelial Cells. Biomacromolecules 3, 1312–1319. 10.1021/bm020074y 12425670

[B110] Zuccotti A. Kuhn S. Johnson S. L. Franz C. Singer W. Hecker D. (2012). Lack of Brain-Derived Neurotrophic Factor Hampers Inner Hair Cell Synapse Physiology, but Protects against Noise-Induced Hearing Loss. J. Neurosci. 32, 8545–8553. 10.1523/JNEUROSCI.1247-12.2012 22723694 PMC6620992

