# Supplementary material for: Development of Neuronal Guidance Fibers for Stimulating Electrodes: Basic Construction and Delivery of a Growth Factor
Source: Front Bioeng Biotechnol. 2022 Jan 24;10:776890. doi: 10.3389/fbioe.2022.776890 (PMC8819688; doi:10.3389/fbioe.2022.776890)
Supplement: Supplementary file 1 [file DataSheet1.docx]

Supplementary Material

**Contents**

Figure S1: Change of pH values of the solutions used in release studies on unmodified (black) and amino-modified fibers (green).

Table S1. Results for survival rates of spiral ganglion neurons after cultivation for two days.





Figure S1: Change of pH values of the solutions used in release studies on unmodified (black) and amino-modified fibers (green).

Table S1. Results for survival rates of spiral ganglion neurons after cultivation for two days.

| sample | | survival rate SGNs / % | | | | | | |  |
| --- | --- | --- | --- | --- | --- | --- | --- | --- | --- |
|  |  |  | supernatant taken after | | | | | |  |
|  |  |  | 1 d | 2 d | 5 d | 7 d | 21 d | 70 d | |
|  | PBS | 5.5  ± 0.7 |  |  |  |  |  |  | |
| controls | medium | 6.1  ± 0.7 |  |  |  |  |  |  | |
|  | BDNF (50 ng∙mL^−1^) | 17.4  ± 1.3 |  |  |  |  |  |  | |
| unmodified  fiber | -HS-BDNF |  | 22.3 ± 5.3 | 18.6 ± 2.0 | 16.1 ± 2.4 | 14.6 ± 2.6 | 12.8 ± 2.7 | 5.1 ± 1.4 | |
|  | -HS |  | 3.9 ± 0.8 | 3.6 ± 0.8 | 6.6 ± 1.5 | 7.4 ± 1.8 | 6.0 ± 1.1 | 0.3 ± 0.2 | |
|  | − |  | 4.2 ± 0.9 | 3.4 ± 0.7 | 3.8 ± 1.2 | 5.1 ± 0.6 | 5.1 ± 0.8 | 0.2 ± 0.1 | |
| amino-modified  fiber | -HS-BDNF |  | 18.6 ± 3.2 | 25.2 ± 5.0 | 14.5 ± 2.2 | 11.4 ± 2.6 | 9.6 ± 1.8 | 8.3 ± 1.9 | |
|  | -HS |  | 4.4 ± 1.0 | 2.9 ± 0.8 | 4.5 ± 1.2 | 5.5 ± 1.3 | 6.0 ± 1.5 | 3.3 ± 1.0 | |
|  | − |  | 2.9 ± 0.8 | 2.9 ± 0.6 | 4.4 ± 0.6 | 3.9 ± 0.7 | 5.0 ± 0.9 | 2.6 ± 0.4 | |
